# Supplementary figures and images for: Variant mutation G215C in SARS-CoV-2 nucleocapsid enhances viral infection via altered genomic encapsidation
Source: PLoS Biol. 2025 Apr 29;23(4):e3003115. doi: 10.1371/journal.pbio.3003115 (PMC12040272; doi:10.1371/journal.pbio.3003115)

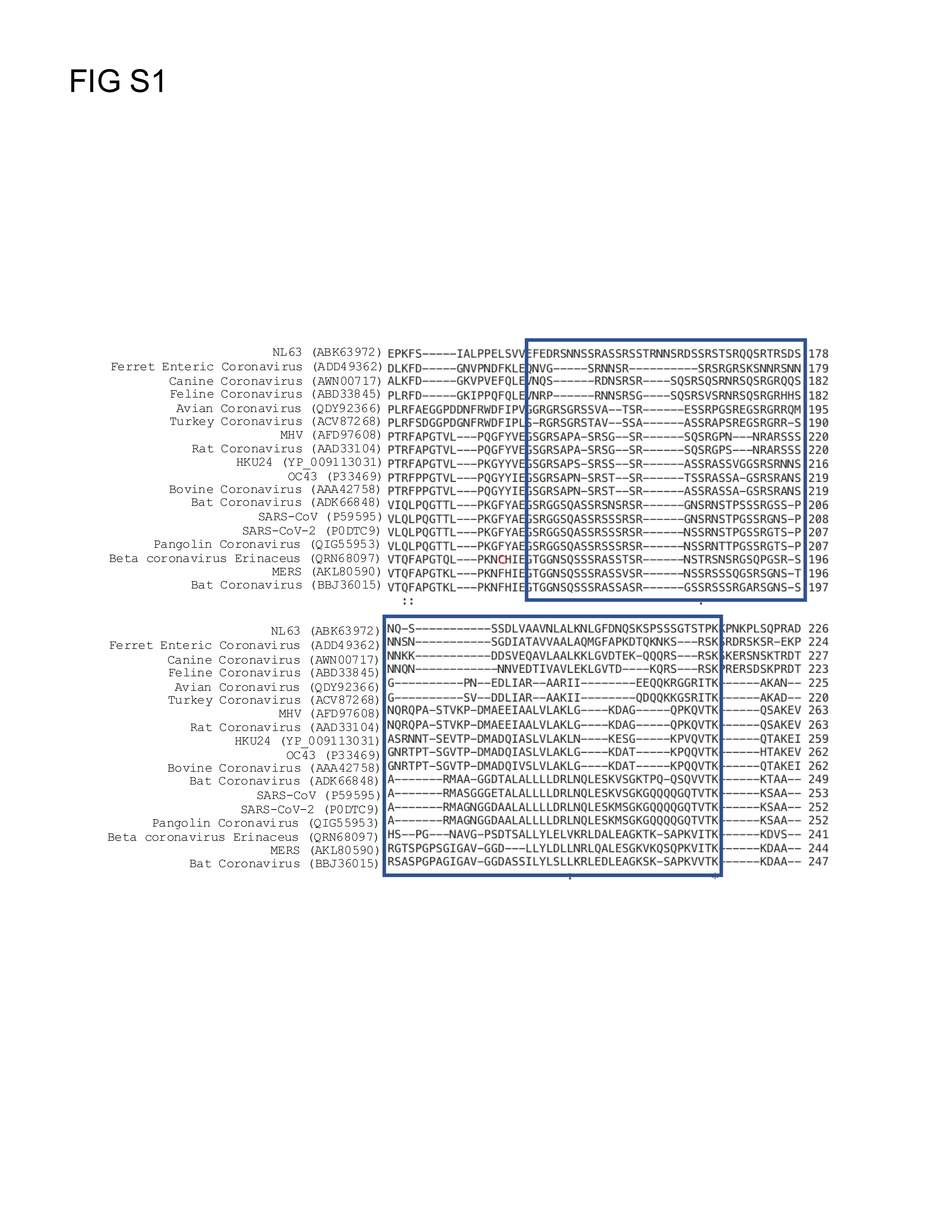

Supplement: S1 Fig — Sequences for the indicated coronavirus nucleocapsid protein sequences were obtained through the NIH Protein Databank and aligned using Clustal Omega from EMBL-EBI (Clustal 0 (1.2.4)). The navy box highlights the linker region (residues 175−247 in SARS-CoV-2). Shown in red is the only cysteine in the displayed sequences, which occurs immediately before the linker region in Erinaceus betacoronavirus. (TIF) [file pbio.3003115.s001.tif]

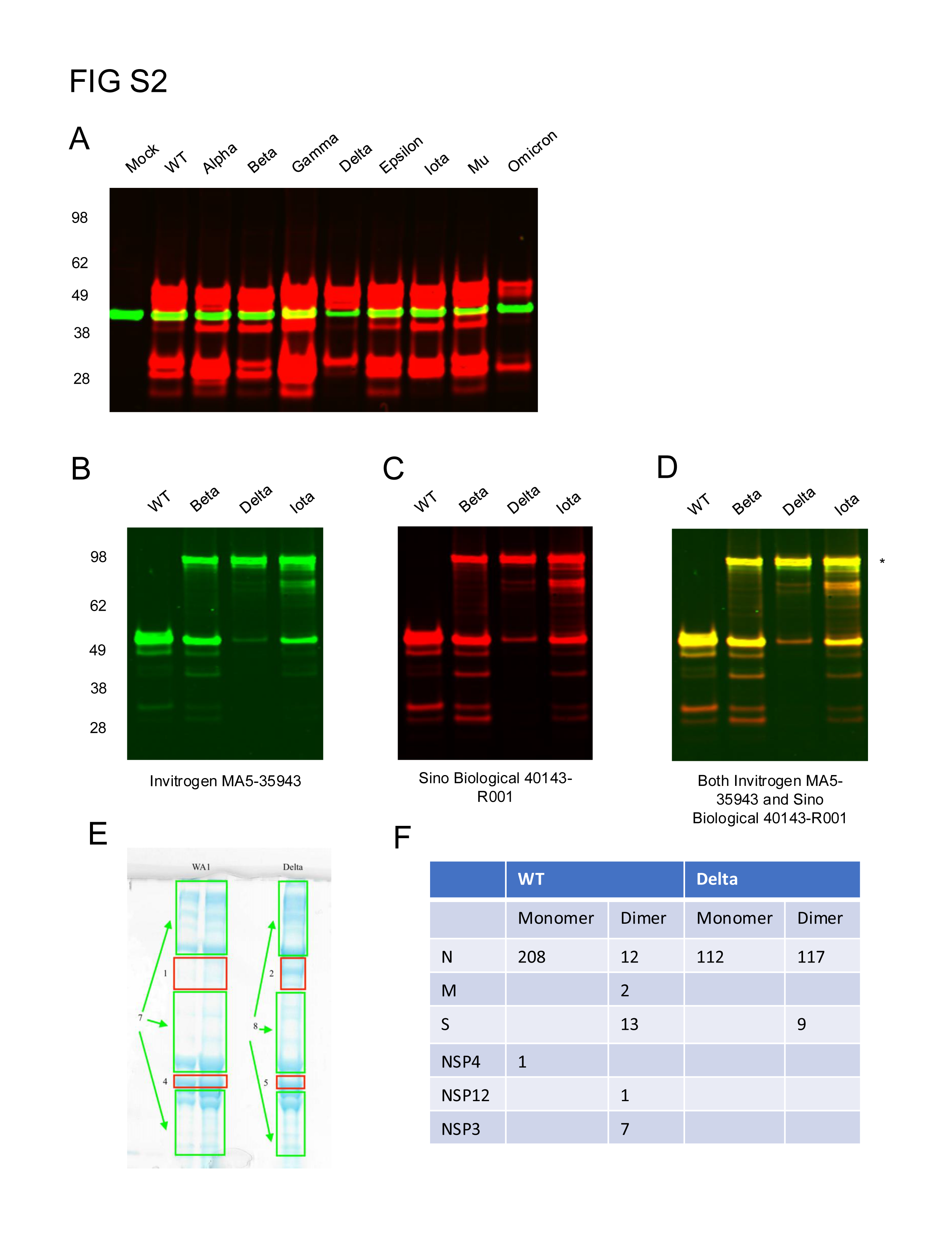

Supplement: S2 Fig — VeroE6-TMPRSS2 cells were infected, or mock infected, with the indicated SARS-CoV-2 variants (or WA1, termed Wild type) at an MOI of 0.01 for 24 h. (A) Cell lysates were harvested under reducing conditions (10-mM DTT) before visualization by SDS-PAGE and western blot using antibodies recognizing SARS-CoV-2 N (red) and β-actin (green). Alternatively, unreduced lysates from cells infected as above were visualized by SDS-PAGE and western blot using two different antibodies recognizing SARS-CoV-2 N: (B) Invitrogen anti-N mouse antibody (MA5-35943, in green) and (C) Sino Biological anti-N rabbit antibody (40143-R001 in red). (D) Lysates were stained and imaged simultaneously with the two anti-N antibodies and the overlay is shown in yellow. Note minor bands that are seen with the Invitrogen but not the Sino-Biological antibody. The MW of the ~100-kDa band observed in Beta, Delta, and Iota samples under non-reducing conditions is indicated by a *. A representative gel from three (A) or two (C) independent biological replicates is shown. (E) Vero-TMPRSS2 cells were infected with WA1 or Delta SARS-CoV-2 at an MOI of 0.01. Twenty-four hpi cells were harvested, lysed, and N was affinity purified using immunoprecipitation. N and associated proteins were run on an SDS-PAGE gel, and bands corresponding to the monomer and dimer were cut and processed for mass spectrometry. (F) The number of peptides for each SARS-CoV-2 viral protein found in the monomer or dimer portion of the gel, for either WT (WA1) or Delta, is shown. The data underlying this Figure can be found in S1 Data and S2 Data. (TIF) [file pbio.3003115.s002.tif]

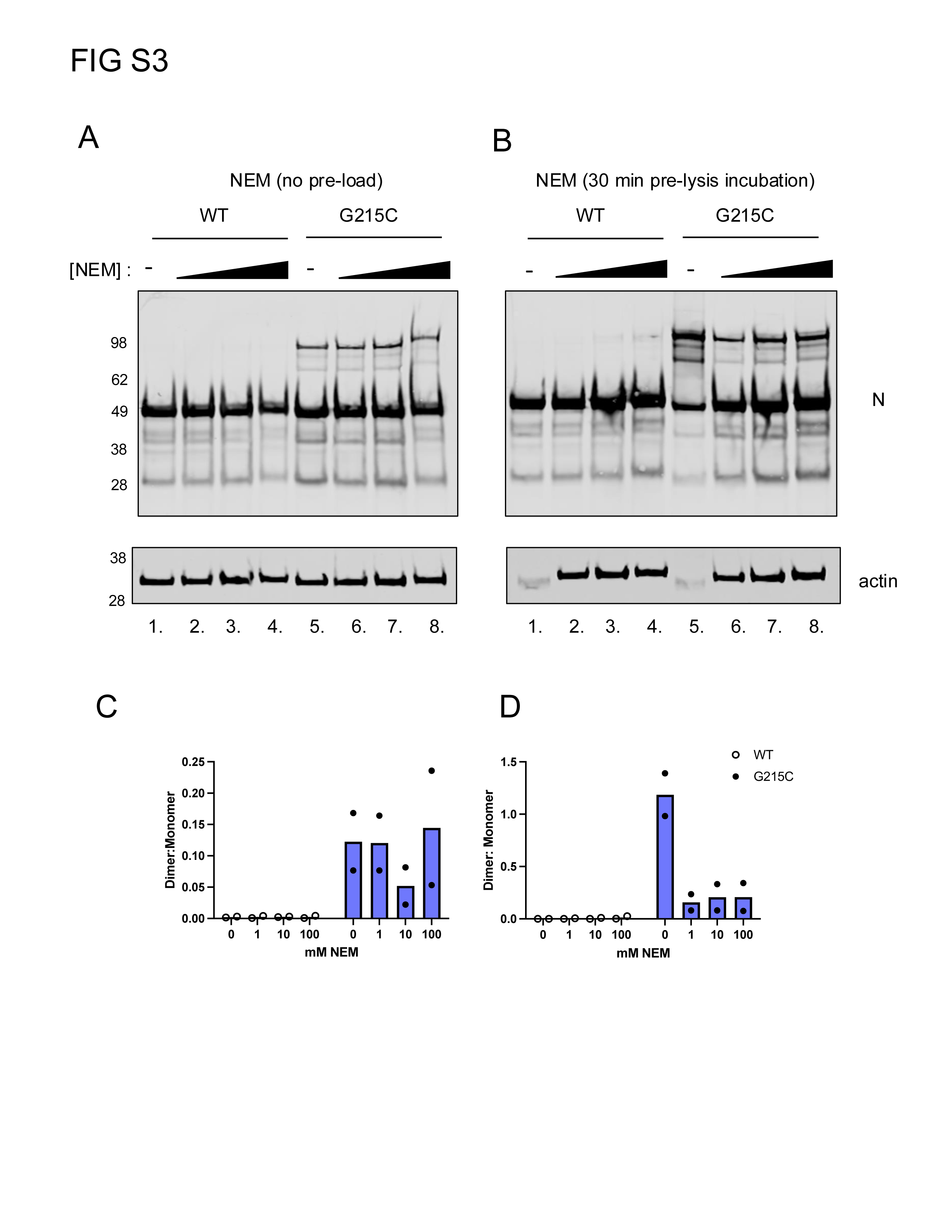

Supplement: S3 Fig — (A) VeroE6-TMPRSS2 cells were infected with WT or the G215C virus at an MOI of 0.01 for 24 h. Cell lysates were harvested in standard triton lysis buffer (lanes 1, 5) or in the presence of increasing concentrations of N-ethylmaleimide (1, 10, 100 mM). (B) A parallel experiment was performed in which cells were pre-treated by incubating for 30 min at 37°C in increasing concentrations (1, 10, 100 mM) of NEM in PBS. Cells were then lysed as in (A), with NEM present in the lysis buffer as well as the pre-incubation. Lysates were visualized by SDS-PAGE and western blot using antibodies to SARS-CoV-2 N and actin. (C and D) Quantification of the N dimer to monomer ratios in (A and B) is shown for two independent experiments. The data underlying this Figure can be found in S1 Data. (TIF) [file pbio.3003115.s003.tif]

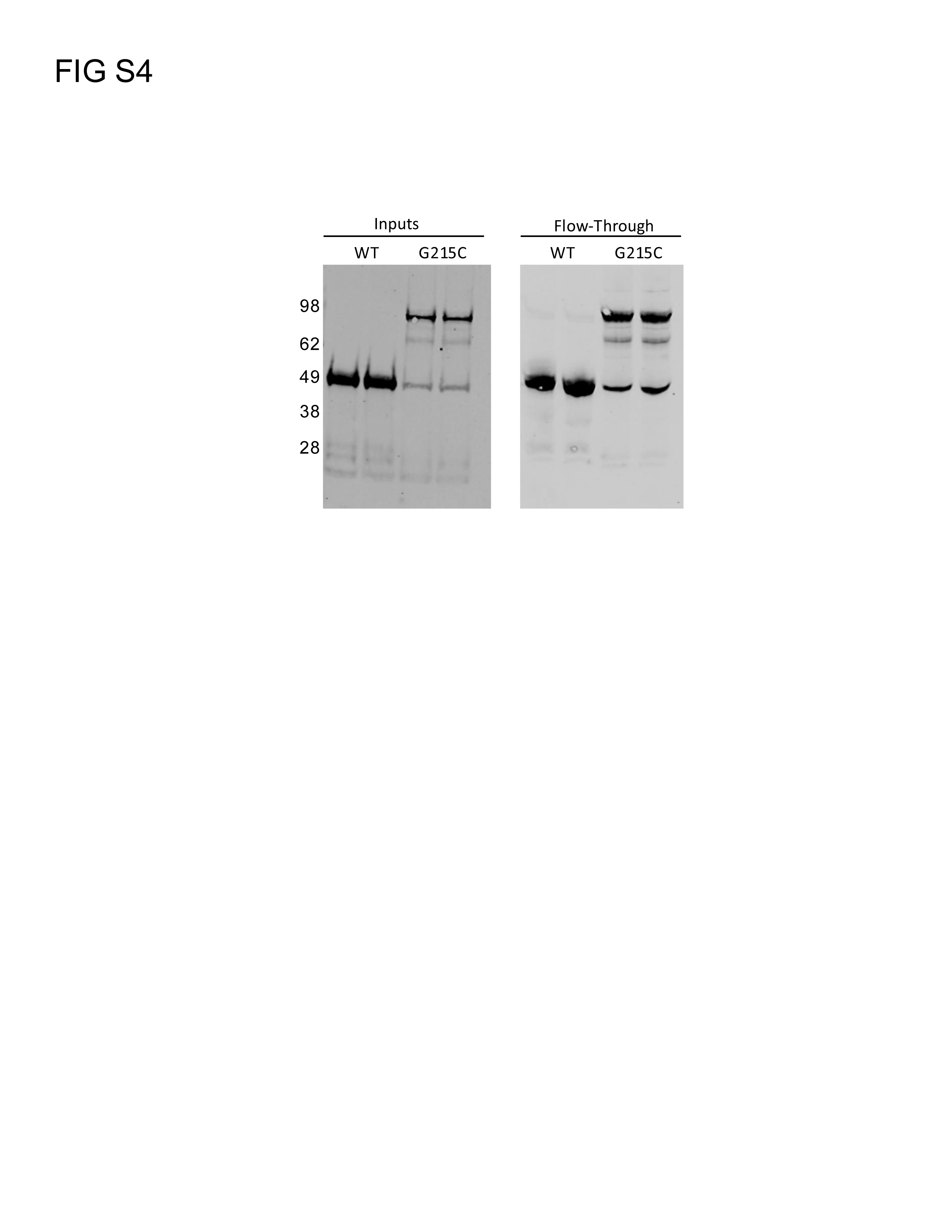

Supplement: S4 Fig — VeroE6-TMPRSS2 cells were infected with WT or the G215C viruses at an MOI of 0.01 for 48 h. Clarified viral supernatants were concentrated by binding to PEG and centrifuging at 10,000G for 30 min at 4°C. Concentrated virus was lysed and nucleocapsid proteins from each flask were affinity purified. Western blots were used to verify equal inputs of N from all conditions (inputs), and saturation of the beads (flow-through). The data underlying this Figure can be found in S1 Data. (TIF) [file pbio.3003115.s004.tif]

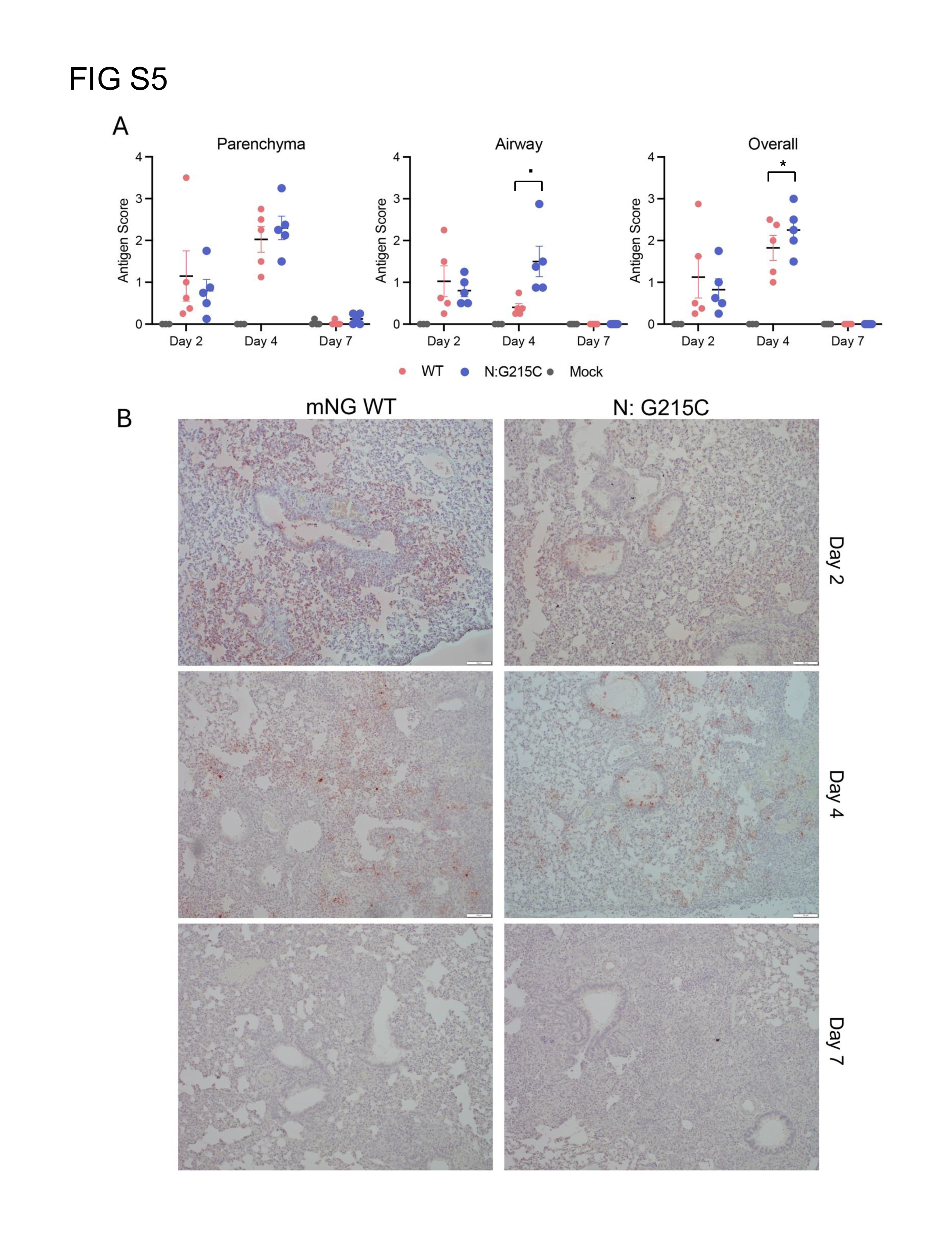

Supplement: S5 Fig — Section of lung tissue from infected hamsters was stained for viral antigen (nucleocapsid). Sections were then blinded and scored on a 4 point scale for parenchyma, airway, and overall staining (A). Each data point represents the average score form two lung section from each hamster in the group (n > 4). Horizontal lines represent the group mean and error bars are ±SD. Significance determined by student T test. (B) Representative images are shown for WT and N:215C infected animals for 2, 4, and 7 dpi. (. [p = 0.05−0.1], * [p < 0.05]). The data underlying this Figure can be found in S1 Data. (TIF) [file pbio.3003115.s005.tif]

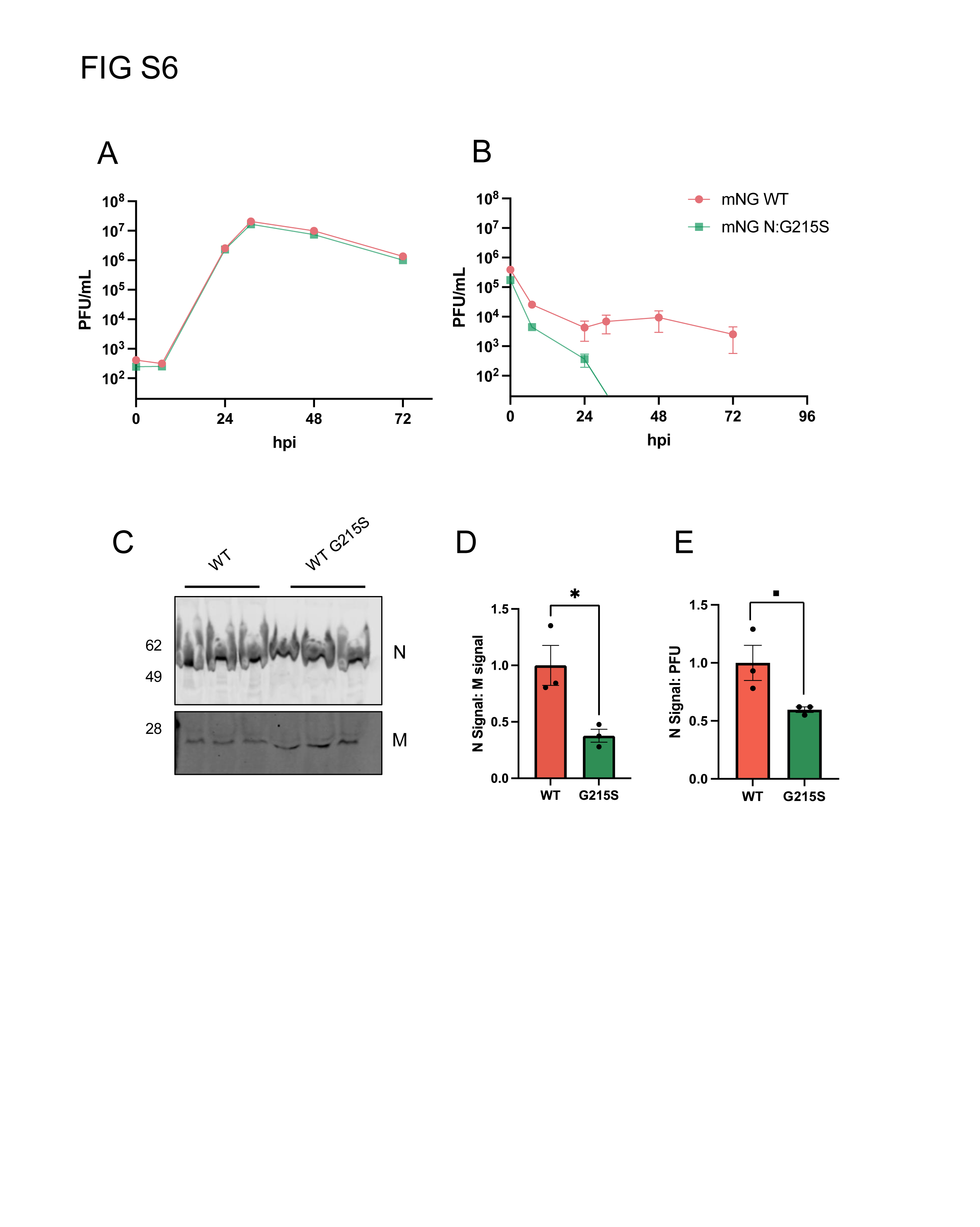

Supplement: S6 Fig — (A) VeroE6-TMPRSS2 cells were infected with the WT or N:G21SC viruses at an MOI of 0.0005 for 1 h, viral supernatants were collected at 8, 24, 32, 48, and 72 h post infection and titered by focus forming assay (FFU; focus forming unit). (B) Human bronchial epithelial cells were infected with WT or N:G215S viruses at an MOI of 0.5 for 1 h, apical washes were collected sequentially from the same well at 8, 24, 32, 48, and 72 h post infection and titered by focus forming assay (FFU). (C) High titer viral stocks of the WT or N:G215S viruses were concentrated by binding to 10% polyethylene glycol then centrifugated at 10,000G for 30 min at 4ºC. Unreduced lysates were collected 24 h post transfection directly from the concentrated viral pellet and N and M were visualized by SDS-PAGE. (D) The ratio of N to M is shown, as well as (E) the ratio of N to FFU. Mean ± SEM is plotted, N = 6 from two biological experiments (A), N = 6 (B), and N = 3 (C–E) is shown. Statistical comparisons were conducted using a two-tailed T test, (. [p = 0.05−0.1], * [p < 0.05]). Limit of detection (LoD) is 20 PFU/mL and the y-axis minimum is set as the LoD. The data underlying this Figure can be found in S1 Data. (TIF) [file pbio.3003115.s006.tif]

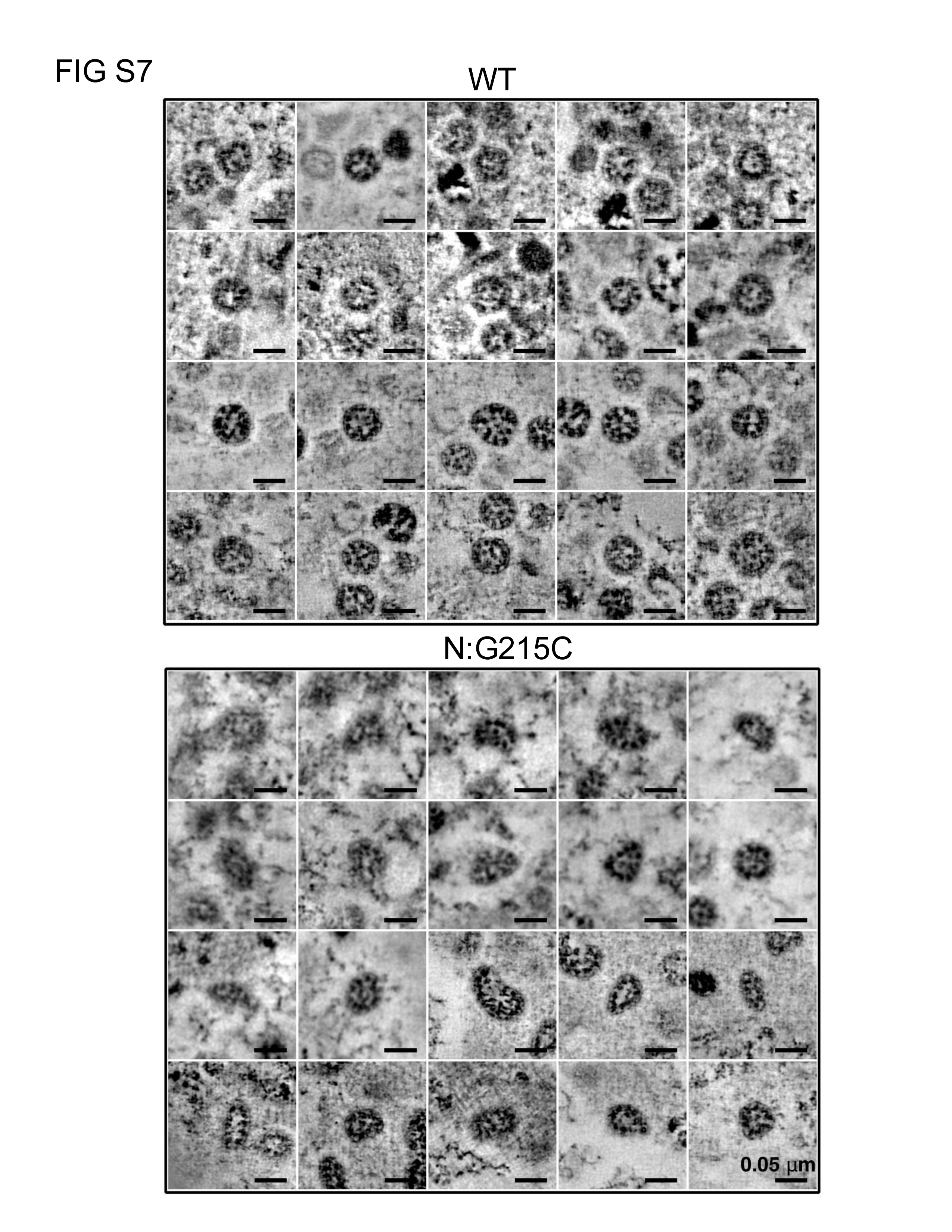

Supplement: S7 Fig — Vero-TMPRSS2 cells were infected with WT or N:G215C viruses at an MOI of 0.1. The following day cells were prepared for electron microscopy by high-pressure freezing and freeze-substitution, then sectioned and imaged by dual-axis electron tomography. Virus-containing exit compartments were located in both samples, and 20 virions that had completely separated from cellular membranes were randomly selected and imaged for each virus. (TIF) [file pbio.3003115.s007.tif]
