# Supplementary material for: Variant mutation G215C in SARS-CoV-2 nucleocapsid enhances viral infection via altered genomic encapsidation
Source: PLoS Biol. 2025 Apr 29;23(4):e3003115. doi: 10.1371/journal.pbio.3003115 (PMC12040272; doi:10.1371/journal.pbio.3003115)
Supplement: S1 Raw Images — (PDF) [file pbio.3003115.s010.pdf]

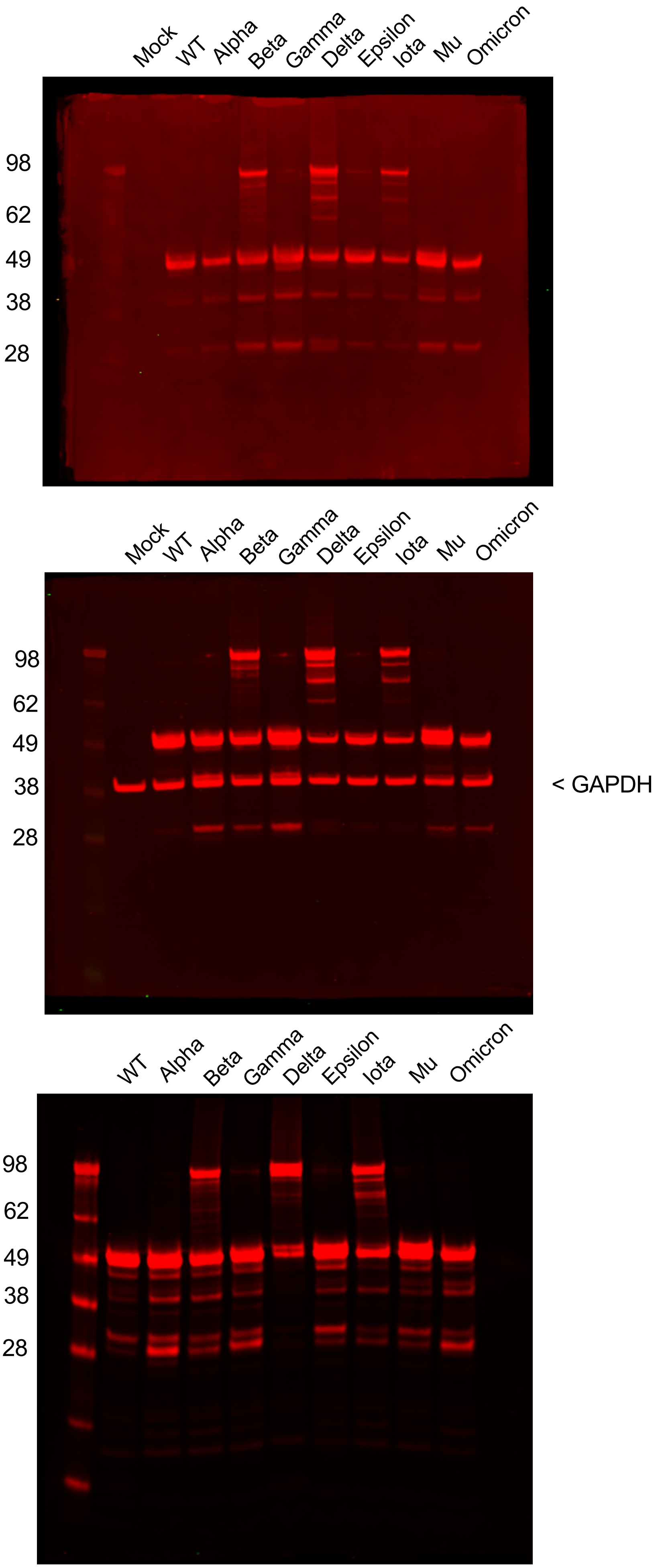

Figure 2A: These membranes were probed with Sino Biological anti-N rabbit antibody (40143-R001 in red). The middle blot was also probed with a mouse GAPDH antibody, before we switched to using a rabbit actin loading control. They were imaged on a Licor Odyssey fluorescent imager.

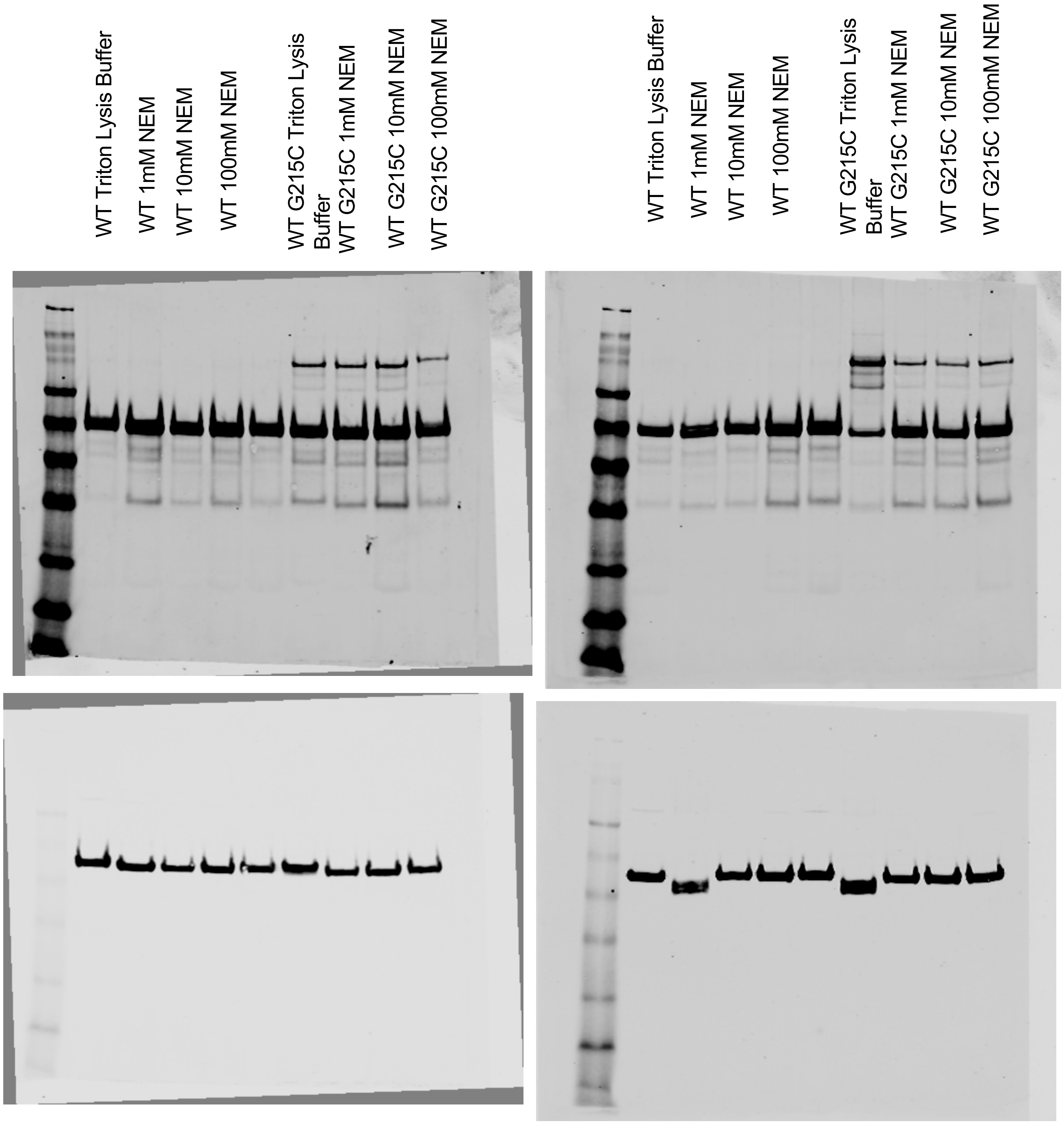

No NEM pre-load

30min Incubation with NEM

Figure 2C (left), 2D (right): These membranes were probed with Sino Biological anti-N rabbit antibody (40143-R001, top) and Novus Biologicals anti-B-actin mouse antibody (NB600-501, bottom). They were imaged on a Licor Odyssey fluorescent imager.

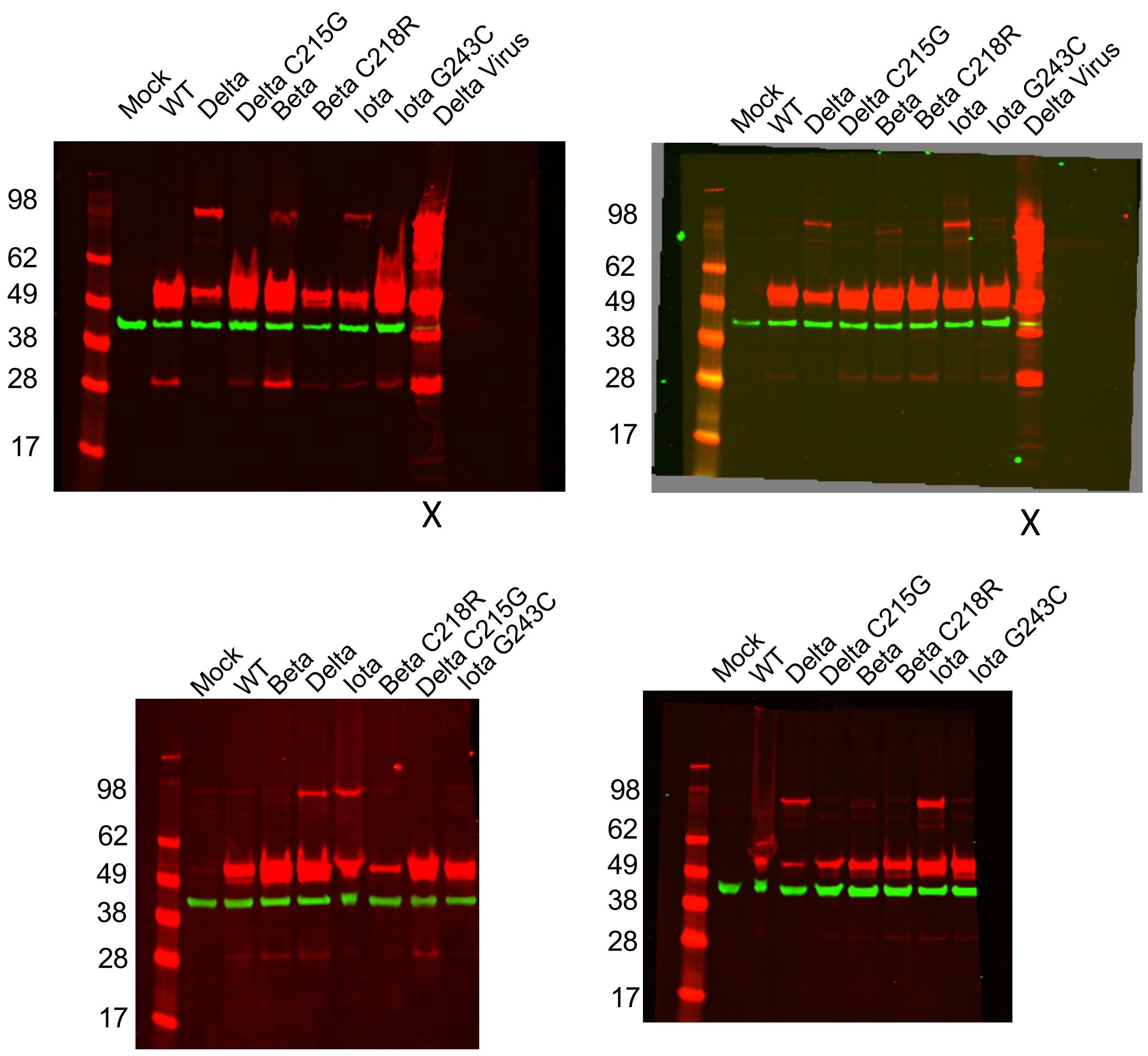

Figure 3 A/B: These membranes were probed with Sino Biological anti-N rabbit antibody (40143-R001 in red) and Novus Biologicals anti-B-actin mouse antibody (NB600-501 in green). They were imaged on a Licor Odyssey fluorescent imager.

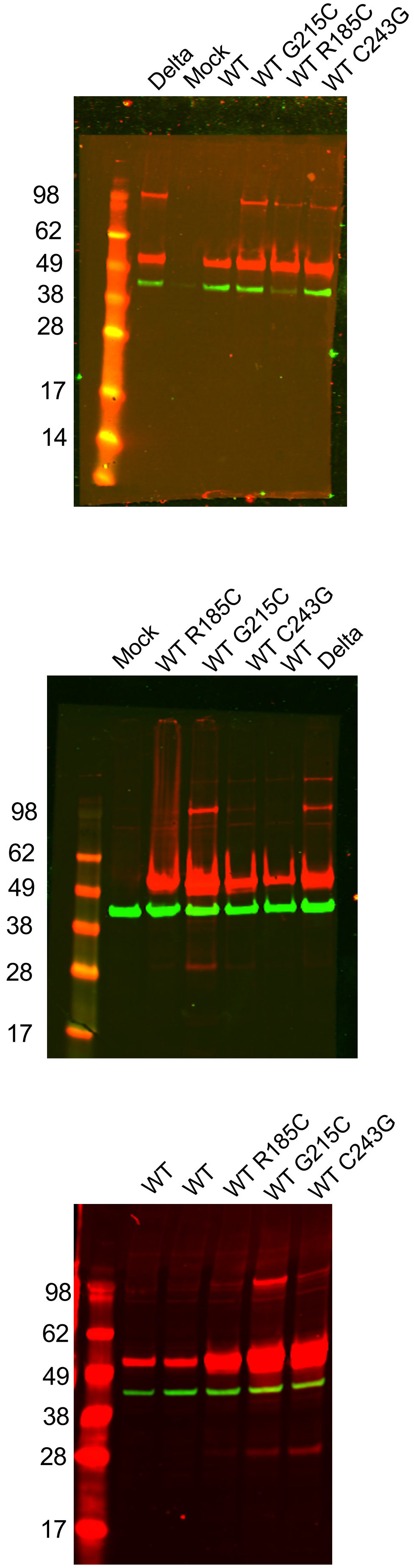

Figure 3 C/D: These membranes were probed with Sino Biological anti-N rabbit antibody (40143-R001 in red) and Novus Biologicals anti-B-actin mouse antibody (NB600-501 in green). They were imaged on a Licor Odyssey fluorescent imager.

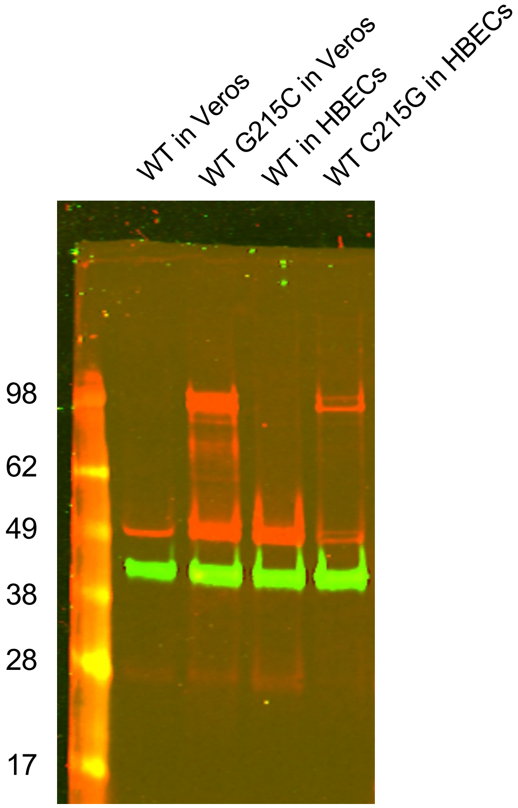

Figure 4 B/D: These membranes were probed with Sino Biological anti-N rabbit antibody (40143-R001 in red) and Novus Biologicals anti-B-actin mouse antibody (NB600-501 in green). They were imaged on a Licor Odyssey fluorescent imager.

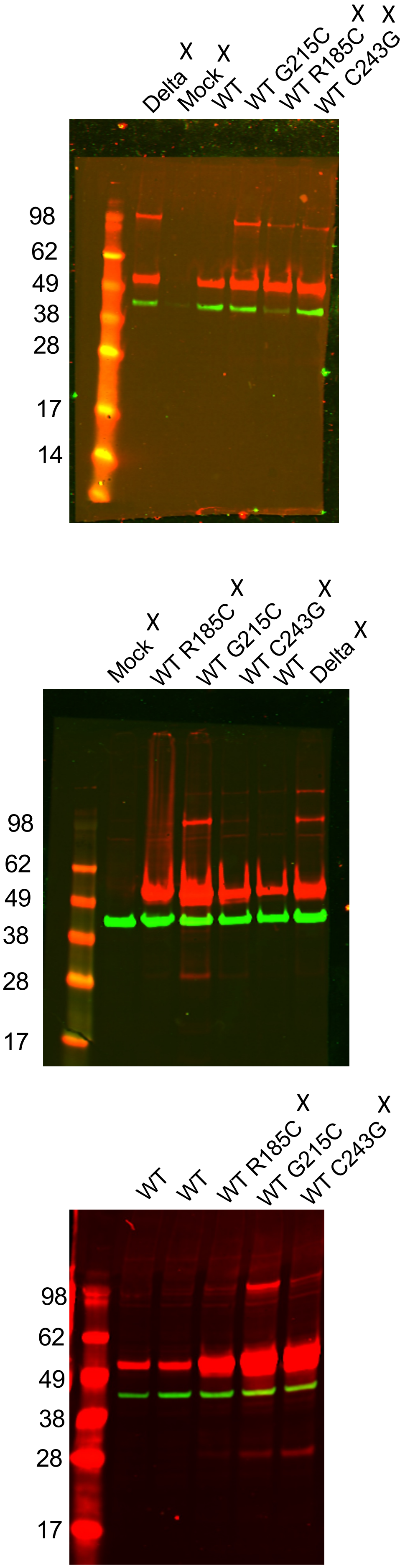

Figure 6 A: These membranes were probed with Sino Biological anti-N rabbit antibody (40143-R001 in red) and Novus Biologicals anti-B-actin mouse antibody (NB600-501 in green). They were imaged on a Licor Odyssey fluorescent imager.

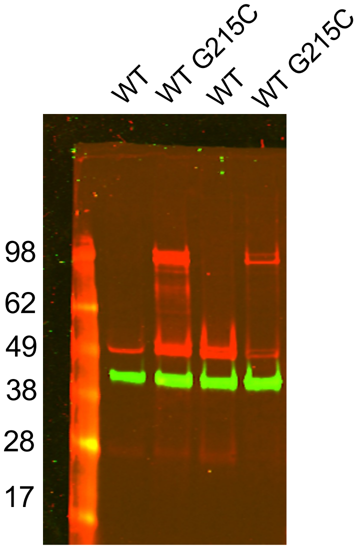

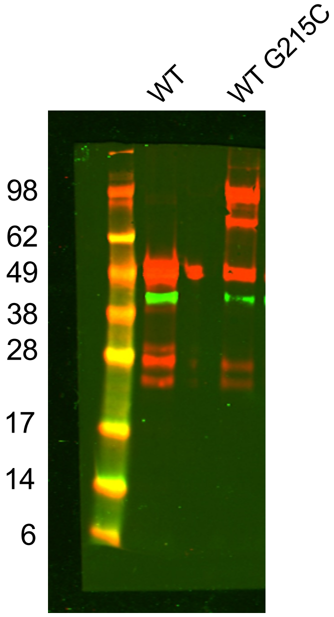

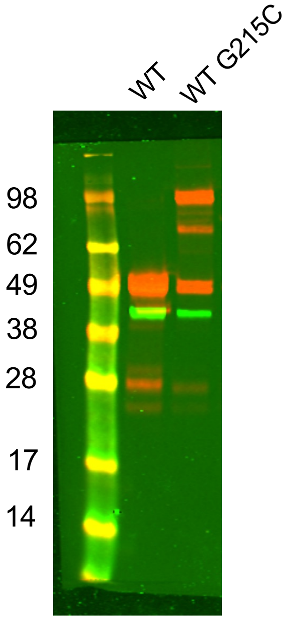

Figure 6 B: These membranes were probed with Sino Biological anti-N rabbit antibody (40143-R001 in red) and Novus Biologicals anti-B-actin mouse antibody (NB600-501 in green). They were imaged on a Licor Odyssey fluorescent imager.

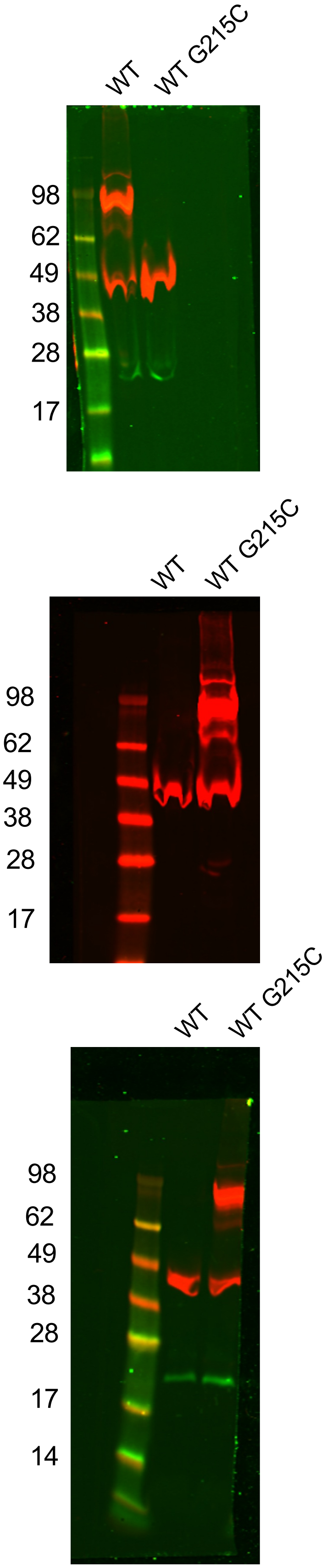

Figure 6 C: These membranes were probed with Sino Biological anti-N rabbit antibody (40143-R001 in red) and Cell Signaling anti-M mouse antibody (E5A8A in green). They were imaged on a Licor Odyssey fluorescent imager.

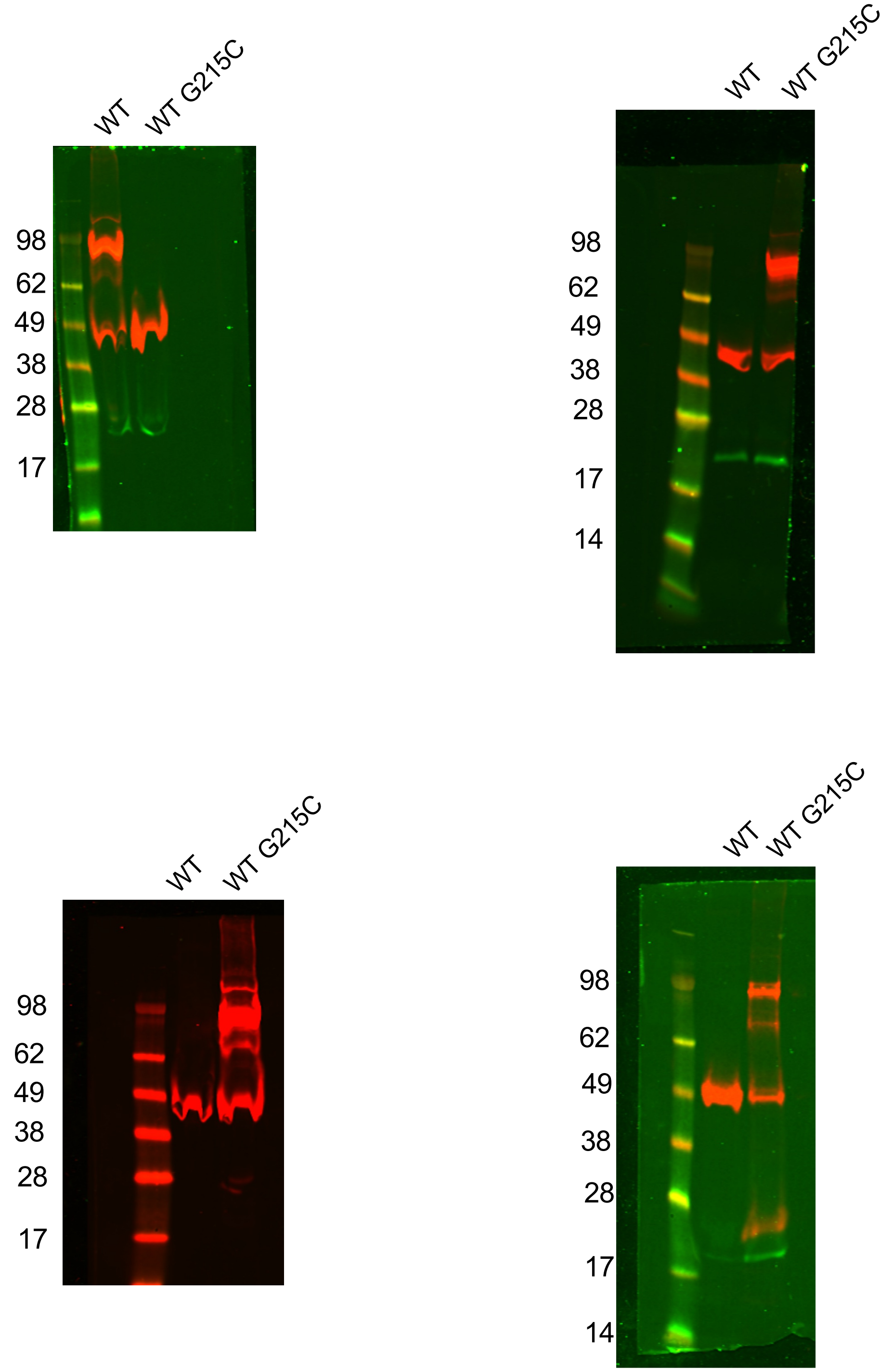

Figure 6 E/F: These membranes were probed with Sino Biological anti-N rabbit antibody (40143-R001 in red) and Cell Signaling anti-M mouse antibody (E5A8A in green). They were imaged on a Licor Odyssey fluorescent imager.

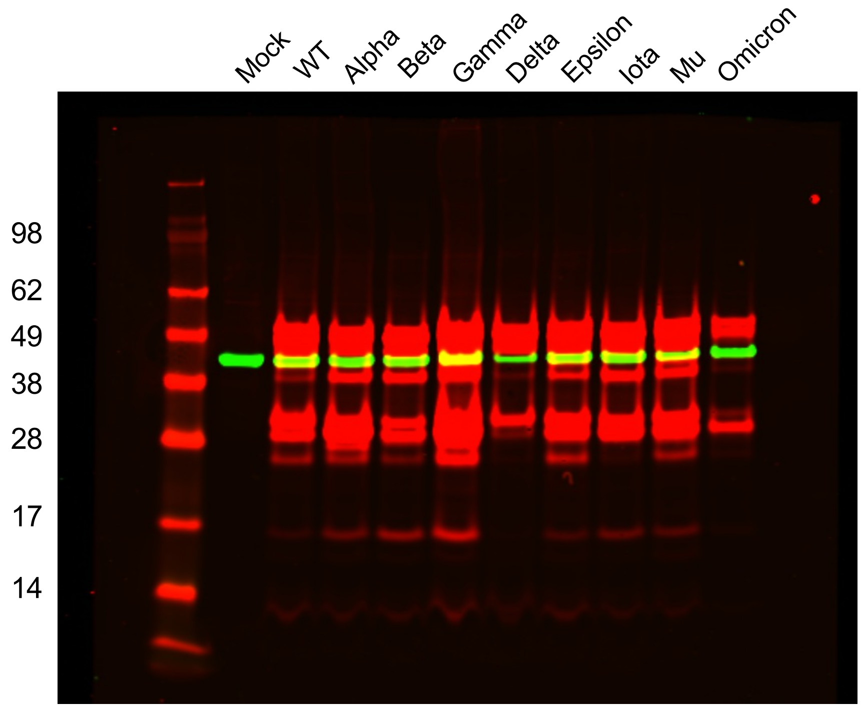

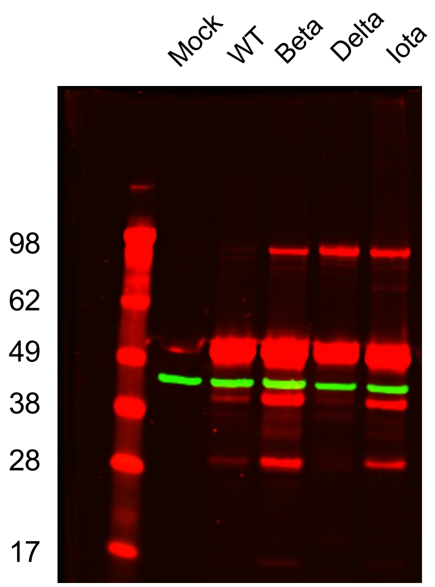

Supplemental 2 A/B: These membranes were probed with Sino Biological anti-N rabbit antibody (40143-R001 in red) and Novus Biologicals anti-B-actin mouse antibody (NB600-501 in green). They were imaged on a Licor Odyssey fluorescent imager.

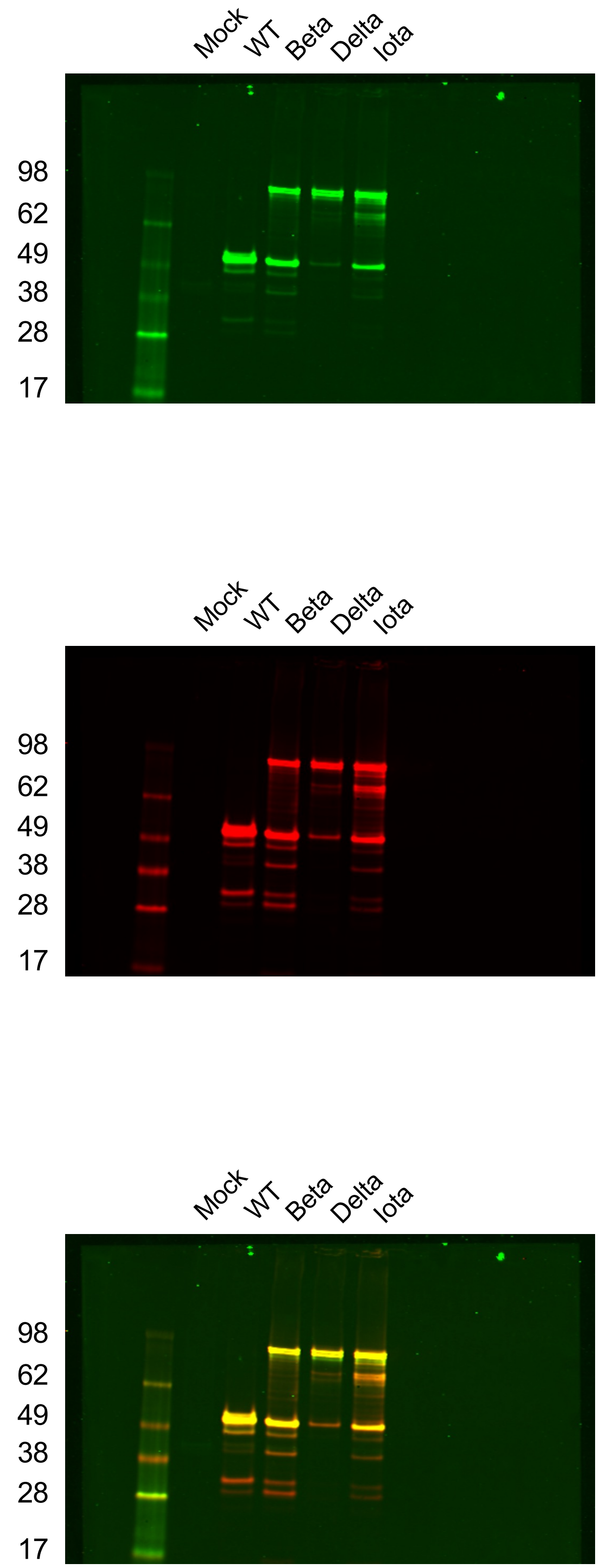

Fig S2B (green),C (red) D (yellow): These blots were probed with Invitrogen mouse anti-NP MA5-35943 (green) and Sino Biological rabbit anti-NP 40143-R001 (red). They were imaged on a Licor Odyssey fluorescent imager.

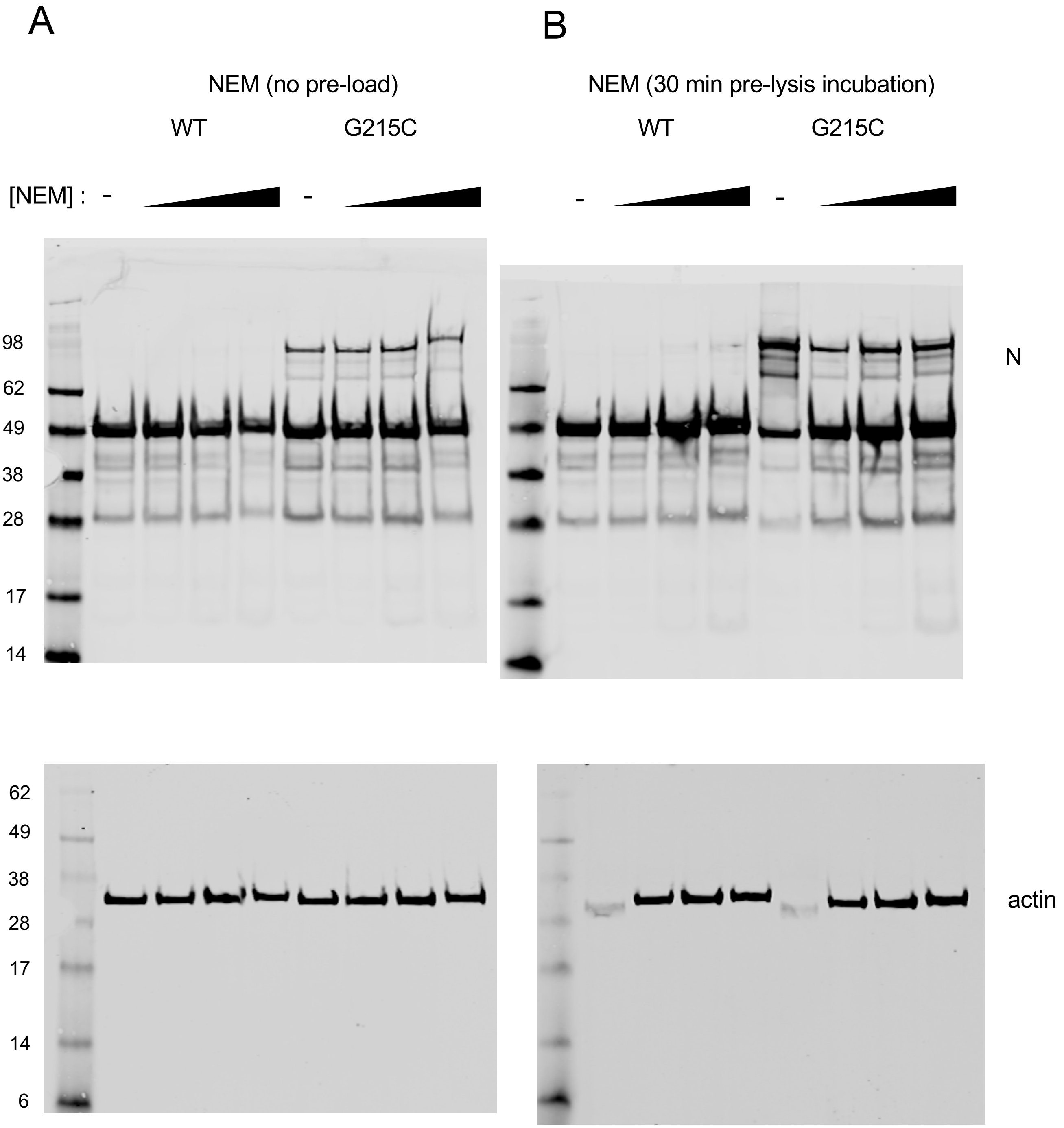

Fig S3: These blots were probed with Sino Biological rabbit anti-NP 40143-R001 (top) and and Novus Biologicals anti-B-actin mouse antibody (NB600-501 bottom). They were imaged on a Licor Odyssey fluorescent imager.

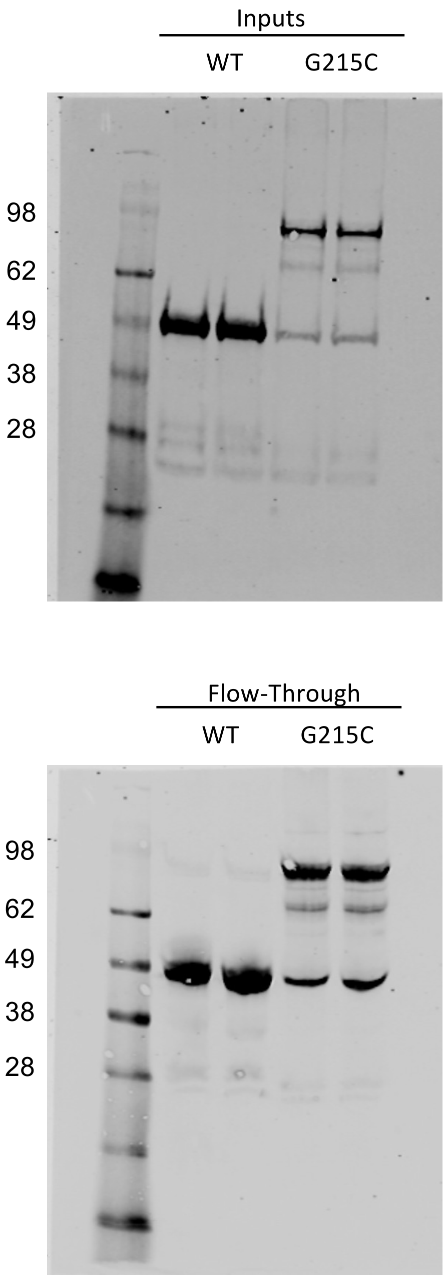

Fig S4: These membranes were probed with Sino Biological anti-N rabbit antibody (40143-R001). They were imaged on a Licor Odyssey fluorescent imager.

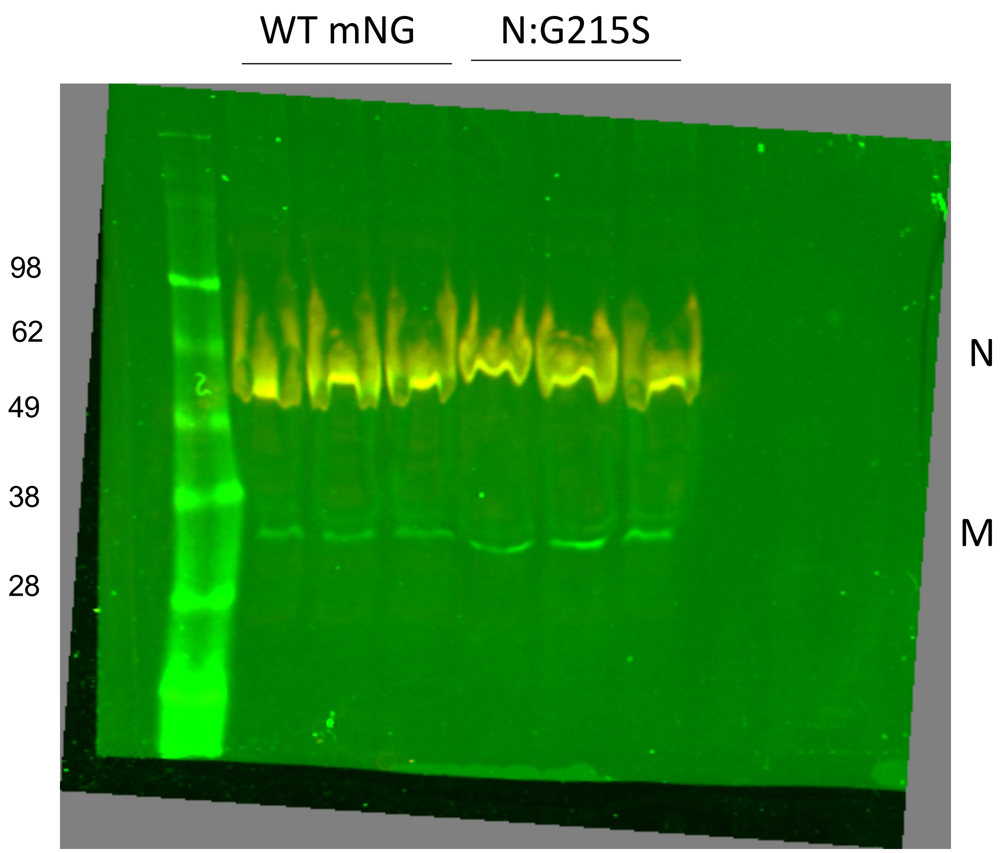

Fig S6: These membranes were probed with Sino Biological anti-N rabbit antibody (40143-R001 in red) and Cell Signaling anti-M mouse antibody (E5A8A in green). (Anti-mouse red secondary was also used on this blot, which is why the N band is yellow). They were imaged on a Licor Odyssey fluorescent imager.

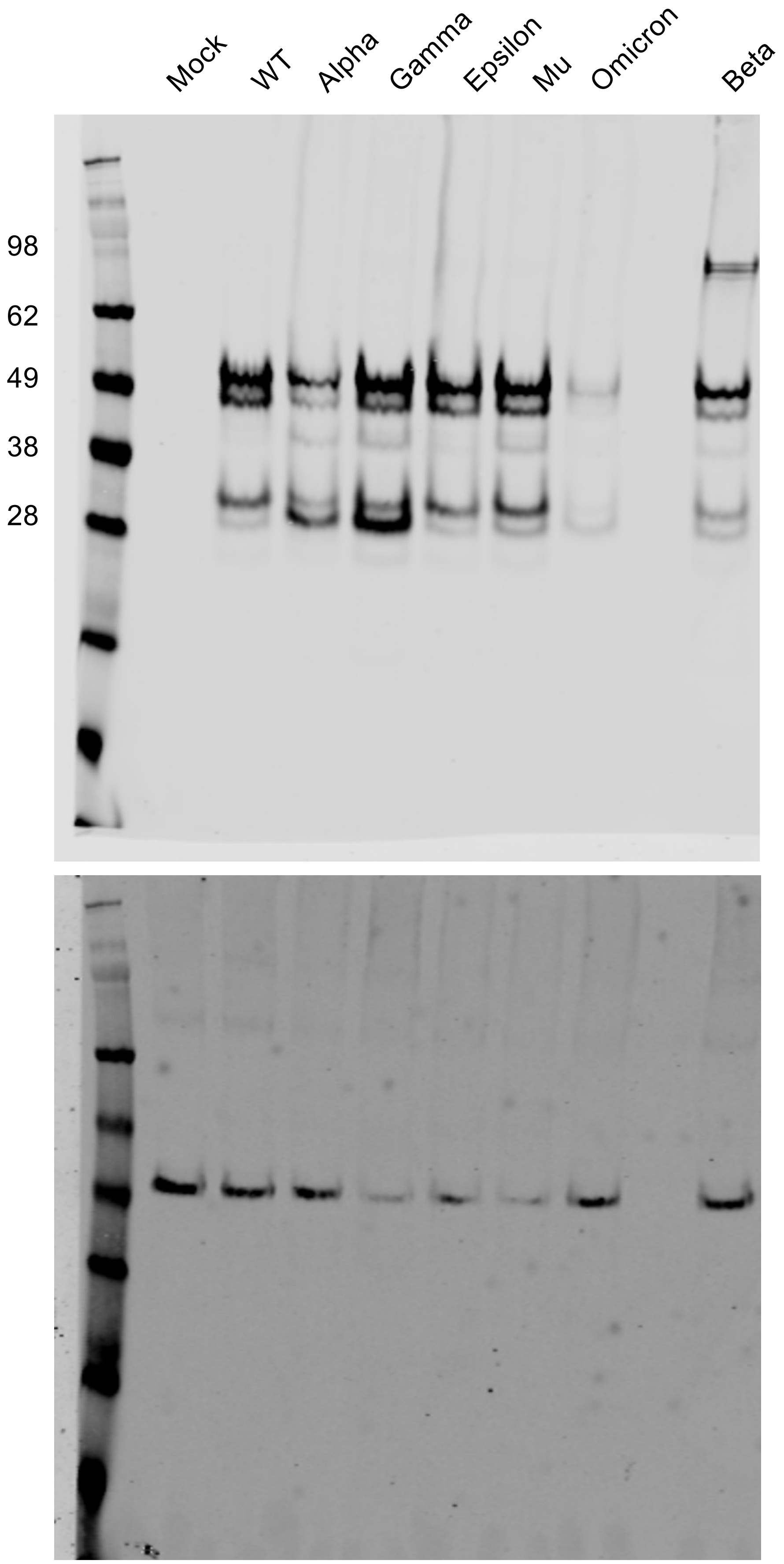

Residual samples from Figure 2 were re-run in a different order to show that stable dimer formation is not seen in the Gamma and Epsilon variants. These membranes were probed with Sino Biological anti-N rabbit antibody (40143-R001 top) and Novus Biologicals anti-B-actin mouse antibody (NB600-501 bottom). They were imaged on a Licor Odyssey fluorescent imager.
