# Supplementary material for: Variant mutation G215C in SARS-CoV-2 nucleocapsid enhances viral infection via altered genomic encapsidation
Source: PLoS Biol. 2025 Apr 29;23(4):e3003115. doi: 10.1371/journal.pbio.3003115 (PMC12040272; doi:10.1371/journal.pbio.3003115)
Supplement: Attachment — Submitted filename: Kubinski_Bruce_prior_reviews.pdf [file pbio.3003115.s011.pdf]

Emily Bruce, PhD, Assistant Professor
Department of Microbiology and Molecular Genetics
University of Vermont College of Medicine
Stafford Hall Room 322
95 Carrigan Dr.
Burlington, VT 05405-0068
Phone: 802-656-9069
Email: Emily.Bruce@med.uvm.edu

The UNIVERSITY of VERMONT

December 13th, 2024

Dear Dr. Madlen Luckner,

Thank you to you and the reviewers for the time and energy you have invested in our manuscript, we believe it is now substantially improved from the original submission. We have completed the experimental revisions requested by the reviewers (NCOMMS-24-16522-T), and the fully revised manuscript is attached. The new data links our *in vitro* observations to the physiological relevance of the G215C mutation seen in the nucleocapsid protein of the SARS-CoV-2 Delta variant.

Specifically, we rescued the N:G215S mutant requested by Reviewer 2 and characterized the growth of this virus in Vero-TMPRSS2 cells, primary differentiated human bronchial epithelial cells (HBEs) and its ability to package N into virions. The N:G215S virus replicated to identical titers in Vero-TMPRSS2 cells (compared to WT), but it under-packaged N in virions, and failed to replicate in primary HBEs. These data clearly show that the covalent disulfide bond is critical for mediating the biological effects (increased viral growth and N packaging) we observed in the G215C mutant (see new Figure S5). As previously discussed, we did not conduct hamster experiments with this virus (due to additional ethical and biosafety approvals), and this has been explained in the point-by-point response.

In addition, we provide new experimental data showing that the covalent interaction mediated by the G215C disulfide bond occurs in infected cells *prior* to lysis and is not an post-lysis artifact (see new Fig 2C, D).

We have also substantially rewritten the manuscript to include additional references and other textual changes requested by the reviewers. We are hopeful that this version of the manuscript now satisfies all three reviewers' concerns and will be acceptable for publication, but please contact me if there are further questions or clarifications!

Sincerely yours,

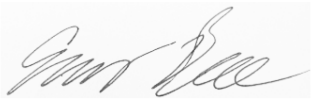

Emily Bruce, Ph.D

Dear Dr Bruce,

Thank you again for submitting your manuscript "Variant mutation in SARS-CoV-2 nucleocapsid enhances viral infection via altered genomic encapsidation" to Nature Communications. We have now received reports from 4 reviewers and, after careful consideration, we have decided to invite a major revision of the manuscript.

As you will see from the reports copied below, the reviewers raise important concerns. We find that these concerns limit the strength of the study, and therefore we ask you to address them with additional work. Without substantial revisions, we will be unlikely to send the paper back to review. In particular, please provide stronger experimental evidence for your conclusion that G215C mutation results in the formation of disulfide bonds in N protein along the lines suggested by reviewer #2. In this context, please provide quantitative data for your SDS-PAGE and Western blotting data (reviewers #2 and #3). Additionally, given the observational nature of your work, we ask you to include the G215S-control experiment in your in vivo work to link the observed increased viral growth to your disulfide bond - hypothesis derived from your in vitro work. Please make sure that these essential revisions and all other requests from our referees are addressed in full in a revised version.

If you feel that you are able to comprehensively address the reviewers' concerns, please provide a point-by-point response to these comments along with your revision. Please show all changes in the manuscript text file with track changes or colour highlighting. If you are unable to address specific reviewer requests or find any points invalid, please explain why in the point-by-point response.

Important: In addition to the above, you must comply with the following editorial requests; we will not be able to proceed with your revised manuscript otherwise. Please also see the Nature Communications formatting instructions, which you may find useful while preparing your revised manuscript.
[information on formatting/ orcid etc removed for space, but I'm happy to forward the original email]
Best regards,

Madlen Luckner
Editor
Nature Communications

REVIEWER COMMENTS

Reviewer #1 (Remarks to the Author):
This work proved the function of the nucleocapsid mutation G215C by comprehensive experiments and indicated that the G215C mutation is capable of packaging more

nucleocapsids per viron and inducing the changes in morphology. The result is meaningful and important. However, thorough revision is required before publication at this moment as my view. More importantly, the discussion part concerning the importance of this mutation needs to be revised. The evolutionary trend of SARS-CoV-2 is towards immune evasion, but there is no evidence in this manuscript supporting the increased immune evasion capability induced by this mutation.
We thank the reviewer for the time and effort they devoted to reviewing our manuscript, we have substantially revised the discussion (see points below). With regards to an evolutionary trend of SARS-CoV-2 towards immune evasion, we are unaware of any data showing that the G215C mutation supports increased immune evasion. The G215C mutation sits outside the recognized T and B cell epitopes driving much current escape from the adaptive immune response (according to the Immune Epitope Database). As we have no evidence at this point to support an increase in immune evasion driven by N:G215C we have decided not to include this statement.

Major and Minor comments:
1, The title did not point out the mutation G215C but the analyses most relevant to G215C.
As the other two reviewers did not raise this as an issue, and 'variant' is easier for the general scientific audience to digest vs 'G215C', we have decided not to change the title at this time.

2, It is said that "the entire viral genome is undergoing evolutionary selection, particularly the central disordered linker region of the nucleocapsid (N) protein." in the abstract. It is better to cite more reference to demonstrate this point.
We have reworded this sentence in the abstract, and as the abstract does not contain references, we have added a substantial number of references to the introduction (see point 3).

3, The manuscript lack thorough review of previews work relevant to the evolution and mutations in the nucleocapsid protein of SARS-CoV-2.
We have substantially re-written the introduction and added additional references. See lines 94-109.
The Alpha, Gamma and Omicron variants contained the R203K/G204R mutations while the Delta (G215C) and Lambda (G214C) variants were characterized by mutations inserting cysteines, all into the flexible linker region (summarized in Nygun et al[12], Kahetran et al[13]). Given the many attributed roles of N, including RNA encapsidation[14], production of viral RNA[15–18], and viral assembly through interactions with M[19–21], mutations within this protein are poised to have large impacts on the viral lifecycle.

The Delta variant, which emerged in mid-2021 possessed the ability to evade prior immunity, increased transmission and altered virulence, and contained a series of novel mutations, including key changes in the spike protein[7]. While the function of the mutations in spike has been intensively studied [4,22,23], the majority of Delta sub-lineages also contained a unique mutation in the nucleocapsid protein which converted a glycine into a cysteine at position 215 (N:G215C)[10,24]. Bioinformatic analysis examining the rate of amino acid substitutions among 2.49 million SARS-CoV-2 sequences deposited in GISAID after the introduction of the Delta variant demonstrated that while most amino acid substitutions are transient, the N:G215C

mutation was maintained over time in GISAID sequence data, independent of concurrent changes in spike or N[10].

4, It is needed to explain why N is a genetic hotspot for mutations in line s 84-86 but a highly conserved protein in lines 89-91.
While the N protein is broadly conserved, amino acid substitutions in SARS CoV-2 variants cluster in the linker region. In order to avoid confusion, we have removed the line about conservation between coronavirus N proteins.

5, It is better to put the sentences in the first paragragh in the results part (lines 109 to 121) to the introduction part, for these sentences is a review of previous finds.
We have removed these sentences and reworked the introduction to include this information.

6, The authors said that "This point-mutation dominated the Delta lineages (Fig. 1A) and bioinformatic analysis suggested that its evolutionary advantage was distinct from co-occurring mutations in spike or nucleocapsid." First, this sentence is ambiguous. Second what bioinformatics analysis suggests the evolutionary advantage needs to be described. Third, what kind of evolutionary advantage needs to be pointed out.
We have included the following line to address the first and second points "*Bioinformatic analysis examining the rate of amino acid substitutions among 2.49 million SARS-CoV-2 sequences deposited in GISAID after the introduction of the Delta variant demonstrated that while most amino acid substitutions are transient, the G215C mutation was maintained over time in GISAID sequence data, independent of concurrent changes in spike or N" (*Line 105-109).

In regards to the third point, it is very difficult to establish what specific kind of advantage underlies a persistent over-representation of a given amino acid mutation in a lineage without experimental studies. Our manuscript is the first *in vivo* study addressing the role of the G215C mutation in N, and provides data to help answer this question.

7, In lines 126-128, it is said that "The introduction of a cysteine in the SARS-CoV-2 nucleocapsid is unique amongst zoonotic Betacoronavirus, as neither SARS-CoV, MERS or other SARS-CoV-2 variants' nucleocapsids contained any cysteines (Fig 1C)". Here, the word unique is not appropriate, considering there should be other nucleocapsid mutations in such situation. If not so, the manuscript should provide more comprehensive and convincing evidences to demonstrate the uniqueness of this mutation.
We have replaced the word "unique" with "unusual" to better reflect that this is one of the only times a cysteine residue has been observed in a coronavirus nucleocapsid protein linker region, we did not intend to imply that this is the only mutation in N that has occurred.

8. the expression "see *" in the line 151 needs to be revised.
We have reworded this sentence, it now reads: *"As predicted, the three variants which contained the novel cysteine residue (Beta, Delta, Iota) produced a second band detected*

at twice the molecular weight of the expected N monomer (Fig 2A, see bands labelled with the asterisk symbol [*]).” (Line 173-175).

9, the sentences “the presence of other viral proteins/RNA and the formation of double membrane vesicles (DMVs) are not required for stably dimerized N formation, and the presence of a cysteine at 215/243 in the Delta/Iota backgrounds is sufficient.” In lines 193-195 is ambiguous and needs to be revised.

We have reworded this sentence, it now reads: *“These data suggest that for Delta and Iota, the presence of other viral proteins/RNA and the formation of double membrane vesicles (DMVs) are not required for stably dimerized N formation. As expressing N protein (in uninfected cells, which lack any additional viral component) results in the formation of a higher molecular weight band, we conclude the presence of a cysteine at 215/243 in the Delta/Iota backgrounds is sufficient to confer the stable N dimer phenotype.*” (Line 226-231)

10, In line 277, the sentence “Interestingly, we noted that the dimer was enriched in extracellular virus” needs to be revised

We have reworded this sentence, it now reads: “*Interestingly, we noted that the ratio of dimer to monomer was particularly high in extracellular virus (Fig 6C, D), though infection (vs transfection in isolation) also appeared to promote formation of the stably dimerized N (Fig 6B vs A).”* (Line 318-320).

11, The reference for the sentence “While SARS-CoV-2 virions lack the rigid organization of a virus with a defined icosahedral capsid, a 50-300% increase of nucleocapsid would seem difficult to accommodate within the standard shape a WT virion” in lines 341 to 343 needs to be shown.

We have included this reference on line 421.

12, The propose that “It is possible that there has not yet been sufficient time for a cysteine-causing mutation to arise at the 214/215 residue of nucleocapsid” in lines 3598-360 lacks evidence. Moreover, the sentence “It is also possible that there are epistatic interactions that mediate the beneficial effect of the G215C mutation that are not present in the Omicron background.” lacks evidence too. It is hard to suggest that G215C will appear in the future based on the provided evidence in this manuscript. In lines 396-399, it is said that “It is possible that the increased oligomerization of N with the G215C mutation drives a greater level of encapsidation that is better able to shield viral RNA from detection by RIG-I and the cellular innate immune response present in primary human lung cells and the hamster model.”, These are also lack evidence. These should be proved by experiments. It is doubtable that this mutation may appear in the future, provided that the distribution of G215C in the global samples’ phylogenetic tree.

These sentences are intentionally included as part of the discussion portion of the manuscript (questions raised by our data/future implications and areas of further research) rather than results (data we show in this manuscript). The paragraph that these sentences are located in begins “*Critical unanswered questions of this study…”* expressly

to highlight that these are important questions raised by our work, but they require further studies (or time) to answer. We intentionally phrased these sentences as possibilities, rather than definite scenarios, because we believe they are important things to consider but are not known facts (also why they are located in the discussion rather than results portion of the manuscript).

13, The analyses of the publicly available genomic data did not includes the data from GISAID. Moreover, the in-silico analyses concerning this mutation is somewhat week.
We chose to include the visualization of the G215C introduction from Taxonium, as this includes more than seven million public sequences sourced from GenBank (i.e. the International Nucleotide Sequence Database Collaboration databases), the China National Center for Bioinformation, and from the COVID-19 Genomics Collaboration UK (COG-UK), and we believe the Taxonium visualization is better suited to this figure. The main point of this figure (that G215C was a mutation introduced early in the Delta lineage, which was maintained during Delta's spread) is also seen in the Nextstrain visualization (which does use GISAID data, to address the reviewer's concern).

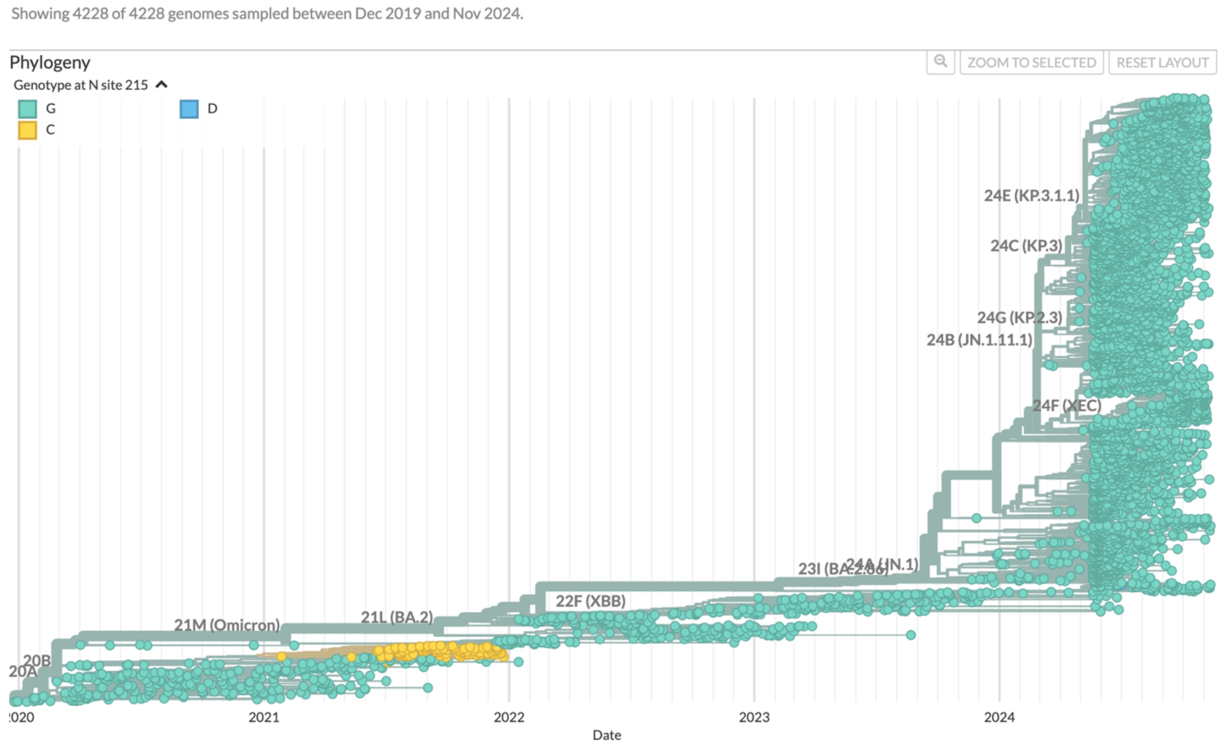

(https://nextstrain.org/ncov/gisaid/global/6m?c=gt-N_215 accessed 11/20/24).

14, bug in line 535.
We have fixed the typo, and "SARS Cov 2" now correctly reads "SARS CoV-2".

15, The format needs to be revised in 629 to 643.

Unfortunately, we are unclear as to the formatting issue the reviewer is referring to and so have not been able to edit these sentences accordingly.

16, the figure legend is not available in some subfigures of Fig5
Unfortunately, we are not sure what the reviewer is referring to. Figure 5 includes panels A-F, and the figure legend also includes a description of panels A through F.

17, the protein structure analyses results in Figure 8 is rough and the visualization needs to be improved.
Figure 8 is intended to be a conceptual framework illustrating that oligomerization is driven by interactions in the linker region that join pairs of dimerized N (and oligomerization status can be 'tuned' by mutations that naturally arose in viral variants), but is not intended as a structural analysis (as it is a cartoon). We have modified the figure legend title and body to reflect this (line 1218-1227).

Reviewer #2 (Remarks to the Author):

This manuscript by Kubinski et.al focuses on a specific mutation (G215C) of the nucleocapsid protein in the SARS-CoV-2 Delta variant and covalent N-N dimer formed through disulfide bonds of the protein mutant. They investigated the impact of the mutation to cysteine using reverse genetics, in vitro SARS-CoV-2 infection with two types of cells and in vivo studies using a hamster model of SARS-CoV-2 infection, observing impact on pathogenicity, viral growth and virion morphology.

Strengths:

The results include in vivo studies, most notable using the Syrian Golden. 230 Hamster model of SARS-CoV-2 infection. The hamsters were inoculated intranasally with either mock, SARS-CoV-2 with N WT, or SARS-CoV-2 with N:G215C. In spite of the kinetic delay, the N:G215C mutant was shown to cause augmented viral replication. The histopathology data indicated the mutant caused higher viral antigen accumulation and more damage in the lung than WT. These results clearly demonstrate the physiological outcomes of the G215C mutation and shed light on the potential influence of this mutation on the severity of the Delta variant. The authors also show the possibility of altered virion structure with the G215C mutation.

Weaknesses:

Major weaknesses of the paper are the lack of mechanistic information, the at best inconclusive assessment of artifactual disulfide linkage of N-protein post-lysis, and a lack of connection that the presumed disulfide links are causing the altered in vivo effects of the G215C mutant. In addition, there is no link to the existing detailed literature on this topic, of which the authors seem to be unaware of, and which might offer alternative explanations for the observed 215C phenotype.

We thank the reviewer for their time and effort in reviewing our manuscript and believe it is substantially strengthened by the revision process. We have substantially re-written portions of the introduction and discussion to include an expanded discussion of relevant literature. We have also included a substantial amount of additional experimental work, as requested by this reviewer. This includes additional mechanistic insight showing an increased association between N and viral RNA in concentrated virions (Fig 6G), proof that the NEM resistant covalent bond occurs prior to sample lysis (Fig 2C, D) and the successful rescue of the N:G215S mutant virus and characterization of viral growth kinetics in Vero-TMPRSS2 cells, primary differentiated human bronchial cells and ability to package N into virions (Fig S5).

Major comments:
1. The SDS-PAGE and Western blotting results are not convincing of the existence of intracellular disulfide-linked N oligomers. The authors carry out control experiments with NEM that are shown in Supplementary Figure S2, intended to support that higher oligomers are not formed post-lysis, arguing that the inability of NEM to suppress the dimer band shows they must have formed pre-lysis, i.e., intracellular. However, to me the data in Figure S2 might show the opposite. The data from Figure S2 should be shown side-by-side with Figure 2 in the main paper to make a comparison easier. But what I visually discern is that the bands with NEM are fainter than those without NEM (comparing Figure S2B with either Figure 2A or Figures S2C/D/E). This leads me to conclude that, apparently, a large fraction of the dimers are indeed formed post-lysis and inhibited by NEM, since otherwise application of NEM would not make a difference. To quantitate this, the authors should carry out an analysis of dimer/monomer ratio of band intensity for the data in Figure S2B to compare with the numbers in Figure 2B. If NEM does suppress the dimer/monomer ratio, as it seems, I am wondering if perhaps a higher concentration of NEM or quicker application would have more completely eliminated the higher oligomer bands (see recommended experiments below).

Related, this control relies on trust in the effectiveness of NEM in completely inhibiting disulfide formation post-lysis. However, the methods indicate that NEM is applied and incubated overnight, after which sample is boiled – I would guess that this might leave ample time for N-N disulfides to form prior to inhibition by NEM. Also, N-protein is prone to aggregation and LLPS, which may inhibit the accessibility of cysteines to NEM. Finally, NEM is not highly effective outside the pH range of 6.5 to 7.5. These issues are problematic and could potentially explain why the dimer bands do not completely disappear in even if hypothetically all disulfides were formed post-lysis.

At present, the data of Figure 2 and S2 appear to be entirely consistent with the idea of post-lysis disulfide formation. If post-lysis disulfide linkage of N-protein is occurring, then the results of Figure 3 from cells transfected with N-protein only would be explainable similarly.

To clarify this point, the authors should do experiments with NEM at different time points and concentrations (including zero NEM) side by side. Furthermore, it should be verified that the pH of the lysate is in the optimal reaction range of pH 6.5 to 7.5 for NEM to be effective – otherwise this would lead to incorrect conclusions.

We have performed the experiment requested by Reviewer 2, and this data is included in the new second half of Figure 2 (see below).

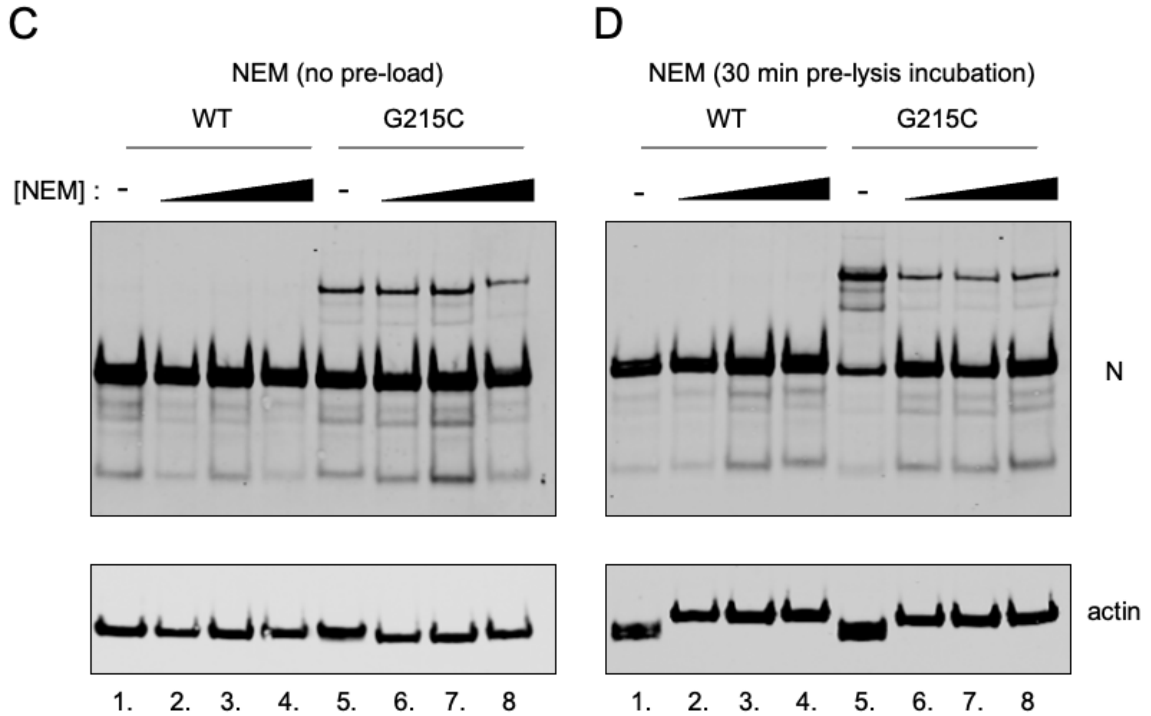

We lysed cells in increasing concentrations of NEM (up to 100 mM, 10-fold higher than the standardly used concentration). As NEM is membrane permeable, we also pre-treated cells in increasing concentrations of NEM for 30 minutes prior to lysis. The result of these experiments is described on lines (267-282) "*To ensure that this disulfide bond truly occurred within infected cells and was not a post-lysis artifact, lysates were harvested in the presence of increasing concentrations of n-ethylmaleimide (NEM), which binds irreversibly to free cysteines and prevents the formation of post-lysis disulfide bonds. Both WT and G215C infected cells, when lysed in our standard triton lysis buffer (Fig 2C, lanes 1 & 5) showed stably dimerized N. When lysed in increasing concentrations of NEM (Fig 2C; 1-10 mM) the stably dimerized form was still present at similar levels to cells lysed without NEM (lanes 7,8 vs 6). To provide an extra level of surety that the disulfide bond was present inside infected cells before lysis, we incubated cells with increasing concentrations of NEM before lysing cells in the same lysis buffer as above, including fresh NEM at the time of lysis (Fig 2D). While a slight reduction in the levels of dimerized N was observed under these conditions, the stably dimerized form remained present even in a ten-fold excess of typical NEM concentrations. This data is consistent with both covalent and non-covalent interactions playing a role in the N-N interaction driven by the cysteine residue at position 215. Given the structural location of these cysteines in the linker region, and the fact that N is known to oligomerize into higher order structures[9,27], we think it is likely that the introduction of this disulfide bond promotes the formation of a tetramer or higher order structure by stabilizing the bond between two non-covalently-bonded dimers.*" (Line 197-213).

To address the reviewer's other points: the buffers (PBS/lysis) we conducted the NEM pre-load/lysis in were a pH of 7.2-7.4, and we boiled samples immediately moving them from BSL3 to BSL2 (post lysis/viral inactivation). We also added 200 U/mL catalase (to quench $H_2 0_2$) and 1 mM DPC-Bio 1 (to block sulfenylated proteins/-SOH) to these lysis buffers, in order to provide increased stringency against the formation of post-lysis disulfide bonds (as recommended by our colleagues who are experts in redox biology- see PMID: 29054072, PMID: 34088769). While there was a slight decrease in the level of dimer seen when samples were pretreated with NEM (new Fig 2D), there was no additional decrease seen with increasing concentrations of NEM. The fact that the higher molecular weight band is still observed in the face of these additional controls suggests to us that the N-N interactions driven by G215C are likely both covalent and non-covalent. As we see higher levels of dimer in infected (vs transfected) cells, and the highest levels of all in extracellular virus, we believe there may be a difference in the protein behavior/interactions seen in the context of *in vitro* (ie, purified protein) vs *in vivo* (ie, virally infected cells, virions) expression. This could explain some of the differences between our findings and those published by the Schuck group that concluded the interactions at G215C are non-covalent. We would like to stress that we believe our results are largely in concordance with those reported by Peter Shuck's laboratory (we both believe that the G215C mutation drives higher order oligomerization of N and increases its affinity for RNA). While we potentially disagree on the degree to which a disulfide bond drives this oligomerization, we are in agreement on the fundamental results that the G215C mutation results in increased oligomerization. We are also in agreement that the G215C mutation increases affinity for RNA (see new Figure 6G). Our studies, while complementary, significantly extend study of this important mutation in the context of fully replicating virus, as well as *in vivo* studies in both primary differentiated human bronchial cells and hamsters.

2. On p 5, the authors report "we discovered that two other variant (Beta & Iota) isolates also contained a cysteine within the N protein". However, neither Beta nor Iota variants do have cysteine mutations among their defining mutations in N (Beta [20H or S.501Y.V2] has a single N mutation T205I, and Iota [21F or 20C/S:484K] has only P199L and M234I) according to covariants.org). Where does this discrepancy come from? Do the authors' other isolates from clinical samples also deviate from commonly understood defining properties of these variants? Since N-protein is highly variable in most positions, random introduction of cysteines is not unexpected, but their significance is not clear.
Our panel of clinical isolates were obtained from US clinical samples (from the University of Washington or the University of Vermont) whose variant identity was determined by NGS (and subsequent lineage calling), for which we had a matching aliquot of virus frozen within the window that would permit isolation of live virus at BSL3. Restrictions on the import of SARS-CoV-2 variants from outside the United States made it unfeasible to include viruses of foreign origin in this study. For variants that did not circulate widely in the United States (ie, Beta, Gamma, Mu) or did so in a limited geographic region for a relatively short time (Iota, Epsilon) there were comparatively few samples available (and it was not possible to recover infectious virus from every clinical specimen). Given the inter-host variation in SARS-CoV-2, we are unsure how one could obtain clinical specimens that contain only the

“commonly understood defining properties” of each variant with no other mutations. We included whole genome sequencing of each of these viruses so other members of the scientific community can interrogate their particular mutation(s) of interest in each variant. As the reviewer speculates, we believe that these two cysteines represent random introductions that were not maintained in the respective lineages, but can nonetheless reveal information about the degree to which certain residues within the linker can support stable dimer formation. We have added the following to the discussion to reflect this: “*Furthermore, while the mutations at 214 and 215 were evolutionarily maintained in the Lambda and Delta lineages, the mutations at 243 and 185 we observed in our Iota and Beta stocks were not maintained in these lineages. The 185 mutation in Beta, while present in our initial viral isolation and p1 stock, reverted back to an R during generation of the p2 stock, suggesting it decreased fitness.*” (Line 435-439)

3. Limitation of the Vero-TMPRSS2 Cells: As shown in Figure 4, after in vitro SARS-CoV-2 infection, “WT and N:G215C viruses grew to identical peak titers, with very similar growth kinetics”. By contrast, experiments using HBECs showed N:G215C virus had improved growth kinetics. The authors explained that this stark difference is because VeroE6 cells are not physiologically relevant, which seems a bit hand-waiving. However, VeroE6 cells were used in many of the experiments in this study. The authors should highlight the limitations of these specific cells in other assays as well.
Vero E6 cells replicate SARS-CoV-2 to very high titers (partially due to their failure to produce type I IFN) and are a crucial tool to investigate fundamental characteristics of a newly generated mutant. While they are not the physiological target of the virus *in vivo*, many of the fundamental processes of viral replication are conserved- and their permissivity makes it possible to conduct experiments (bulk concentration, electron microscopy, proteomics) that would be difficult if not impossible to carry out in primary cells. We have added the following clarifying statements to the results section:

- *Due to their permissiveness and ability to support high levels of viral replication we first characterized the activity of the newly rescued virus in Vero-TMPRSS2 cells.* (Line 251-252)
- *Next, as VeroE6 cells are African green monkey kidney cells that lack a functional type I IFN system, we examined viral growth kinetics in primary differentiated human bronchial epithelial cells (HBECs), grown in transwells on an air-liquid interface (ALI).* (Line 260-262)
- *Due to the difficulty in transfecting primary differentiated bronchial cells and lack of sufficient material for concentration, we performed these experiments in Vero-TMPRSS2 cells.* (Line 312-314).

4. Regarding the unique oblong morphology: On page13 the authors state “This finding is particularly interesting in light of recent observations that the virions of a clinical Delta isolate retain the spherical shape and radius size found in the ancestral SARS-CoV-2 lineage37, suggesting that additional mutations in the Delta lineage have the effect of counteracting the phenotype”. The authors should confirm this phenomenon using other

cells as well. Did the authors examine the morphology of the viruses produced in the hamster experiments, which would provide clear information on this?
Unfortunately due to the inactivation protocols in place at the BSL-3/BSL-4 facility at the University of Texas Medical Branch, we are unable to complete the necessary steps to preserve tissue for successful thin section electron microscopy experiments while abiding by biosafety regulations for that facility.

5. The authors greatly overstate the originality of their observation and the manuscript lacks adequate references to prior work. That coronavirus N-protein can exist as disulfide-linked multimers has been shown for MHV several decades ago (Robbins et al., 1986. RNA-binding proteins of MHV: detection of monomeric and multimeric N protein with an RNA overlay- protein blot assay. Virology 150:402-410), and should be cited. Further, the authors claim "We have identified a novel cysteine..." suggests a discovery by the authors, where in fact the G215C mutation has been prominently pointed out as a driver for SARS-CoV-2 Delta-variant infectivity by other laboratories previously, and extensive structural work has been carried out in atomistic detail on the effect of this mutation including the promotion of intermolecular disulfide bonds as well as significant enhancement of non-covalent oligomerization (PNAS Nexus 1 (2022) pgac049, Science Advances 9 (2023) adg6473 and references cited therein). Similarly, the authors have missed recent data reported on exacerbated viral assembly induced by the G215C nucleocapsid mutation in a virus-like particle assay (bioRxiv 10.1101/2023.11.22.568361, which is Nucleic Acids Research doi 10.1093/nar/gkae256).
We have removed the sentence "We have identified a novel cysteine.." and substantially re-written the introduction. We apologize for not citing Zhao et al, NAR, 2024, this manuscript was not published until after our study was already under review but we have added a detailed discussion of this paper to the introduction (line 110-134).
"This introduction of a cysteine residue within the nucleocapsid is unusual amongst pandemic-causing betacoronaviruses and has major structural implications as it could result in the production of a stable N-N dimer linked by a disulfide bond. Here we describe the impact of this novel cysteine within the N protein and the role it plays in viral replication and particle formation. We demonstrate that this cysteine promoted N oligomerization by stabilizing a covalent N-N interaction that resolves as a dimer on standard non-reducing SDS-PAGE gels. While higher order structures composed of N multimers that disappear upon reduction have been previously reported for both mouse hepatitis virus (MHV)[25], Bovine Coronavirus, and OC43[26] (in each case the higher order multimer appears to run as a trimer of N, rather a dimer as observed here). Furthermore, none of these viruses contain a cysteine in the linker region, suggesting that the N-multimerization previously observed for other coronaviruses may be fundamentally different the process driving SARS-CoV-2 nucleocapsid oligomerization.

Several studies published during the time this work was in preparation and review have also elegantly examined the role of the G215C mutation in nucleocapsid function. Early characterization of purified Delta nucleocapsid protein revealed that it was more compact and less discorded than the ancestral WA1 protein, and that the canonical Delta mutation G215C increased N self-association, shifting the protein from a primarily dimeric

to tetrameric state via disulfide cross links of noncovalent dimers, while increasing its association with nucleic acids[10]*. Subsequent studies from the Schuck group identified a highly conserved transient helix structure in a leucine-region of the N linker that can cooperatively assemble into coiled-coil trimers, tetramers and higher order oligomers in a process that is further enhanced by binding to nucleic acid*[27]*. The N:G215C mutation stabilizes this helical state (and thus enhanced self-association), which in turn enhanced activity in a virus like particle (VLP) assay measuring relative expression of a reporter gene packaged into VLPs created by transfecting the four main structural proteins into HEK 293T cells*[28]*."*

6. Exemplifying the disconnect with the literature, p 5, line 136 the authors state: "Since the introduction of a cysteine residue would allow for the formation of a new disulfide-bonded N-N dimer complex, we predicted that this mutation could have major impacts on the secondary, tertiary and/or quaternary protein structure of the nucleocapsid protein." Looking at the literature, it has been unambiguously shown in biophysical studies that G215C does indeed allow higher oligomers to form (above cited PNAS Nexus), both covalent as well as non-covalent, and even that G215C impacts the secondary structure promoting helical structure. Therefore, the hypothetical posture of the authors seems disingenuous, even more so as the authors actually they cite this work, although solely for its bioinformatics work.

We agree that much is now known to suggest that G215C allows higher order oligomers to form, but we began this project before any of these papers was published. We observed the higher weight molecular weight bands of N in our Beta, Iota and Delta clinical isolates in early 2022, ~two months before the Zhao et al, 2022 manuscript was published in PNAS Nexus on May 16, 2022. Furthermore, the first author of this paper was an undergraduate student for the majority of this project (she started graduate school this fall), which meant our rate of progress was slower than that of larger groups. Our writing reflects our thinking and the hypotheses governing our experimental rationale, given the state of what was known at the time we were conducting the experiments. Nonetheless, we have modified our manuscript to include more detailed discussion of concurrent studies relating to this work (see reply above, to point #6). The Zhao PNAS Nexus paper was originally cited and discussed in the discussion in the context of the linker region driving oligomerization, but we have added additional references and to this portion of the discussion. See lines 384-391:

"This theory is supported by recent work showing that conserved but transient helices in the leucine-rich sequence of the linker can assemble cooperatively into higher order structures (including trimers, tetramers and higher oligomers), and stabilizing this structure results in an increase in oligomerization as well as VLP activity[10,27,28]*. While these studies were largely conducted on purified protein (either the linker alone, or larger portions of nucleocapsid), our results here suggest that this process is occurring in bona fide viral infection, when N will need to successfully carry out a series of different functions to drive a complete cycle of viral replication."*

7. The in vivo results of the G215C mutant on the WA1 background are very interesting. In order to demonstrate whether intermolecular N-N disulfide bonds are indeed responsible for these, we would propose control experiments with G215S, with serine at 215 serving as an analogue of 215C with similar physicochemical properties, and with similar ability to enhance the non-covalent coiled-coil oligomerization of N in the adjacent leucine-rich region. As shown recently, it is the non-covalent oligomerization of this region that is critically important for viral packaging (bioRxiv 10.1101/2023.11.22.568361; Nucleic Acids Research doi 10.1093/nar/gkae256), and this multimerization can be strongly enhanced by nearby mutations including 215C and 215S (Science Advances 9 (2023) adg6473). If the authors carried out control experiments with G215S, they could assess whether this produces a similar in vivo phenotype as G215C. If it does not then this would significantly strengthen the authors' hypothesis that N-N disulfide bonds are responsible.

As requested, we rescued the N:G215S mutant (see new figure S5 below). Due to the lack of a phenotype in HBEs (as well as biosafety concerns) we did not perform experiments in the hamster model with the G215S mutant, as we (and the Institutional Animal Care and Use Committee) feel this does not represent an ethical use of experimental animals. We

FIG S5

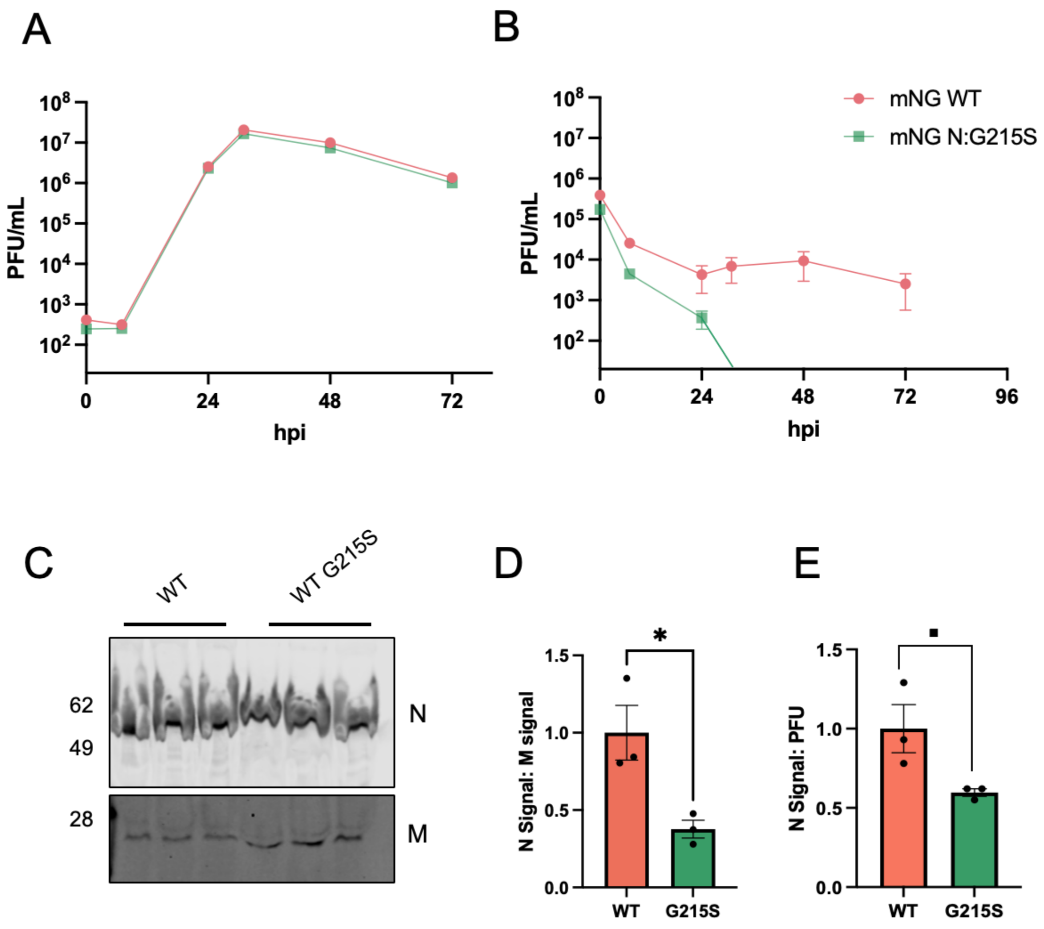

describe the results on lines 334-353:

“Parallel work published while this study was under preparation and review suggested that the G215C mutation enhanced the non-covalent coiled-coil oligomerization of N critical for packaging. These studies proposed that the oligomerization was driven primarily by non-covalent (vs covalent) interactions, with a serine able to functionally substitute for a cysteine at this position[27,28]. To evaluate the impact of a serine at this position, we generated a SARS-CoV-2 N:G215S mutant and characterized its replication and the packaging of N into virions (Fig S5). While the N:G215S virus grew with identical titers to WT in Vero-TMPRSS2 cells (Fig S5A), the mutant failed to replicate in primary differentiated human bronchial epithelial (HBE) cells (Fig S5B). Next, we concentrated extracellular virus from Vero-TMPRSS2 cells to examine the levels of N and M seen in virions (Fig S5C). The G215S virions had reduced nucleocapsid compared to both a separate structural protein (Fig S5D; M) and infectious units (Fig S5E; PFU). The fact that G215S underpackages N in virions suggests that covalent interactions at the G215C position may allow N oligomerization and encapsidation, even in the context of fully replicating virus. However, the failure of the G215S virus to replicate in primary differentiated HBEs reflects an additional role for covalent N-N interactions mediated by a cysteine lost in the context of a serine at that position. Alternatively, the 215S mutation may disrupt kinase and 14-3-3 recognition motifs necessary to balance N phosphorylation and regulate its function.[8,35–38]. Importantly, the covalent interaction of the G215C mutation allows for increased N oligomerization and encapsidation without the loss of replication capacity observed in the G215S mutant.”

8. There are other processes that could be impacted by G215C unrelated to oligomerization (covalent or non-covalent), for example, modulation by the G215C (or maybe G215S) mutant of intra-molecular contacts recently discovered by NMR (bioRxiv 10.1101/2024.02.22.579423) or alteration of essential contacts of the nearby leucine-rich linker helix to NSP3 (Science Advances 2022 10.1126/sciadv.abm4034). Therefore, while the observed in vivo phenotypes described in the present manuscript are very interesting, their correlation with putative covalent N-N dimers is tenuous. The authors should comment on these possibilities.

We agree that the implications of the G215C mutation on the interaction of N with NSP3 is of great interest (we had previously discussed the 2022 Science Advances paper above on lines 633-644). We are somewhat in disagreement however, that the interaction between the N linker and NSP3 is necessarily independent of oligomerization. In the context of viral replication it is possible that the nucleocapsid interacts with NSP3 at one time/phase of the life cycle, and with other nucleocapsid proteins to mediate oligomerization at a different time (both via the linker region). Our data suggests a model in which the G215C mutation *increases* encapsidation/oligomerization at the possible expense of N-NSP3 binding (a theory for which we have preliminary data, but that work is the focus of a separate student’s project so we are not including here). We have expanded the discussion to include these points.

“Curiously, our work suggests a model in which stabilization of N-N interactions that occur through the linker region could reduce the ability of N to bind to NSP3, with the N-N linker associated oligomers and N-NSP3 binding scenarios being mutually exclusive (Figure 8).

Further work is needed to reconcile this theory with the finding in this study that the G215C mutation increases encapsidation." (line 470-472)

"Our data indicates that the stably dimerized form of N is packaged at higher levels into mature virions, this effect may be partially due to a decreased ability of cellular kinases to access the key phosphorylation sites. It is also possible that the stably dimerized form of N resulting from the G215C mutation slows the rate of uncoating during viral entry, both by increasing affinity of N for RNA and by preventing hyperphosphorylation by the cellular kinases SRPK1/GSK-3/CK-1 which drive a conformational switch in N that abolishes interaction with RNA[44]." (Line 402-408)

Minor points

1) P 3 line 85/90: It is stated that the N-protein is a mutational hotspot, but later that it is highly conserved. At face value these statements seem contradictory without clarification.
While the N protein is broadly conserved, amino acid substitutions in SARS CoV-2 variants cluster in the linker region. In order to avoid confusion, we have removed the line about conservation between coronavirus N proteins.

2) Line 155 reads "…was not seen by SDS-PAGE gel/western blot for viruses that lacked cysteines …" but in Figure 2A, there are faint but significant bands at the N dimer position also for Gamma and Epsilon. How do the authors interpret these bands?
The faint bands seen at the dimer location in Gamma/Epsilon in Figure 2A are very small amounts of protein that spilled from one lane to the next during loading. We re-ran a set of (limited) remaining samples not expected to form a dimer (including Alpha and Gamma), alongside a positive control (Beta) and confirmed that (when loaded with an empty lane in between) the Alpha and Gamma variants do not make any visible N dimer (see below).

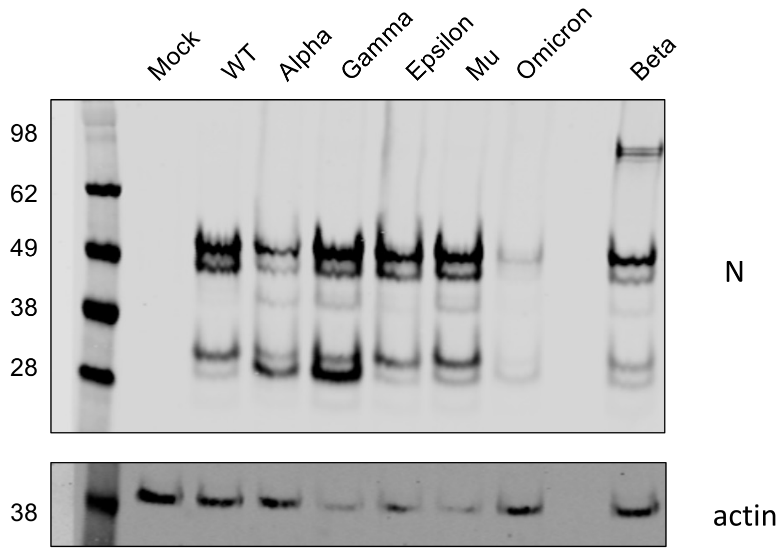

3) Also regarding the SDS-PAGE gel: Most lanes show several intense lower molecular weight bands – could proteolytic activity impair the detection of N oligomers? If so, could proteolytic activity be different for the different variants?

While we also assume that the lower molecular weight bands are likely to be proteolytic cleavage events, we do not believe they will impede the detection of N oligomers, as we see a similar pattern of lower molecular weight bands that run below the putative dimerized N. We have also noticed potential changes in presumed proteolytic activity between variants, and intend to investigate this further in future studies.

4) I am surprised that the mass spectroscopy experiments were not utilized to report directly on peptides with disulfide-linked linker fragments, which presumably should have been formed after digestion of the dimer band.
Unfortunately we did not get good coverage of this portion of the protein with our tryptic digest, so we are not able to answer this question with our current dataset.

5) page 8, the authors state “we transfected HEK-293T cells with either WT N, or WT N containing a single point mutation (R185C, G215C, and G234C). When visualized via western blot in non-reducing conditions, only the construct which contained the Delta G215C mutation could produce the stabilized dimer, suggesting that this mutation is both necessary and sufficient for stable dimer formation (Fig 3C,D).” Can the authors comment on why the other two mutations did not show the stabilized dimer?
The other two mutations did form a stabilized dimer in some experiments (see the full uncropped gels provided with this submission, also pasted below). To better reflect that data we have reworded this sentence to read *“When visualized via western blot in non-reducing conditions, the construct which contained the Delta G215C mutation most robustly produced the stabilized dimer across biological replicates, suggesting that this mutation is both necessary and sufficient for stable dimer formation (Fig 3C,D).”* Line 241-244.

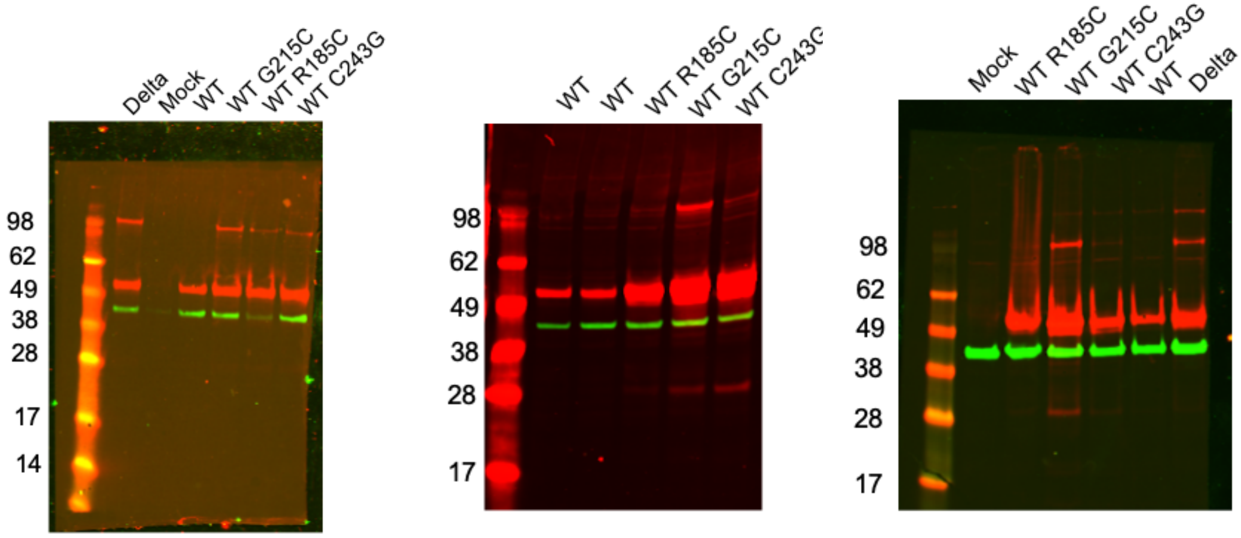

6) The MW shown on the SDS-PAGE is ~47 kDa, and the disulfide bonded-dimer band migrates around 98 kDa. The MW of the N protein monomer based on amino acid

sequence (such as sequence of GenBank: QIC53221.1) is lower, at 45.6 kDa. Can the authors comment on this difference, or how is the precision limited?
The N monomer runs close to the 49 kDa marker on 4-12% SDS-PAGE gels, but I do not believe we can clearly resolve the difference between ~47 kDa and 45.6 kDa with our current molecular weight ladder and SDS-PAGE gel equipment.

7) The observation of variability of the dimer/monomer ratio dependent on the localization of the nucleocapsid samples adds a valuable dimension to this work. Based on recent work on phosphorylation presenting a functional switch of SARS-CoV-2 N-protein the question arises to what extent the different samples examined are phosphorylated. It might be outside the authors' intended scope, but if gels are available they might be subjected to mass spec analysis to quantify the level of phosphorylation, and to establish how this correlates with the N oligomerization. The authors speculate on this in the Discussion, but may actually have an opportunity to provide data on this.
We agree this is an interesting and significant line of inquiry, however we do not have good coverage of tryptic fragments in the relevant portion of the linker region and so we are unable to answer this question with our current dataset (as we were not able to collect peptides that span the region of the N linker that is phosphorylated with our current digestion/mass-spec protocol).

8) Line 293 and Figure 7: micromolar(?) diameter – probably micrometer (lower case 'm')
Thank you for finding this typo, we have made the appropriate correction.

Reviewer #3 (Remarks to the Author):

The authors detail the effects of cysteine point mutations within the N protein linker, present in certain Sars-CoV-2 variants of concern, using both in vitro and in vivo measurements. The in vitro assays clearly show that these cysteines, especially the 215C present in the Delta variant, promote stable N protein dimerization, attributable to disulfide bonds forming between cysteine residues. Single point substitutions, in which cysteine is added to WT N protein, also shows effects, though less pronounced than comparisons of full variant sequences. Overall, the manuscript makes a compelling case providing a biophysical explanation for these effects. We present minor potential edits below and would recommend publication with these adequately addressed.
We thank the reviewer for the time and effort they devoted to reviewing our manuscript. We have made all the edits suggested (see below).

The representative gels present very conclusive results that do not always seem reflective of the total data set. For instance, Fig 3C shows a clear dimer band for G215C, but the average results shown in 3D are not statistically significant due to a single below average dimer ratio data point. This raises the question whether that western blot didn't show a clear dimer band, or if the monomer band had increased intensity and reduced the ratio despite a clear dimer band appearing. The fact that each variant has one conspicuous low ratio experiment also makes one wonder if these results were all from the same gel, such

that the G215C has a more pronounced dimer band in each biological replicate, but this trend is obscured by the averaging. Finally, the reporting of dimer:monomer ratio is a convenient way to normalize results but may obscure trends in overall N protein expression. This is of particular interest as the authors later show (Fig 6) that the G215C mutation increases viral packaging. For instance, the 3C gel shows both larger monomer and dimer bands for the cysteine mutants, as compared to WT. Is this a consistent trend? Because of this wide variation in gel results, it may be useful to add a supplementary figure that contains images of all the gels represented in these measurements.
We have included images of the full uncropped images of all gels with the revised manuscript. The three gels that were quantified to produce the data graphed in Figure 3D all clearly show the presence of a dimer band in the G215C construct. We agree with the reviewer that variations in expression/monomer levels likely explains variations in the magnitude of the effect, but the presence of the dimer is still clearly seen in each of the experiments that led to this figure. Likewise, we have included full uncropped images of all blots shown in the manuscript, as well as the full uncropped images of all biological replicates from which quantification was used to generate graphs of monomer:dimer ratios.

Markers of statistical significance are not displayed in figures 2 and 3 as they are used in later figures, and these would be helpful to add.
We have performed statistical analysis and added the appropriate markers of statistical significance to Figures 2 and 3, as requested.

Reviewer #4 (Remarks to the Author):

I co-reviewed this manuscript with one of the reviewers who provided the listed reports. This is part of the Nature Communications initiative to facilitate training in peer review and to provide appropriate recognition for Early Career Researchers who co-review manuscripts.
Thank you to Reviewer #4 for their feedback in improving our manuscript.

Emily Bruce, PhD, Assistant Professor
Department of Microbiology and Molecular Genetics
University of Vermont College of Medicine
Stafford Hall Room 322
95 Carrigan Dr.
Burlington, VT 05405-0068
Phone: 802-656-9069
Email: Emily.Bruce@med.uvm.edu

January 29th, 2025

Dear Dr. Luckner,

Please find the revision of our manuscript (NCOMMS-24-16522A) attached.

As requested, we have modified the manuscript title (Reviewer 1 request). In addition, we have provided a second western blot of NEM treatment (see new Fig S3A/B) as well as quantification of both experiments (see Fig S3 C/D) as requested by Reviewer 2. We also provide context of why additional mass spectrometry experiments are both outside the scope of the current manuscript and would fail to provide definitive answers to the reviewer questions. Finally, we have tempered our conclusions to clarify that the data is consistent with both covalent and non-covalent N-N interactions at the G215C position, as requested (see lines 214-216 and 383-387). Together, we believe these additions and changes suitably address the major concerns of the reviewers.

Overall, the major findings in the manuscript are the functional impacts of the G215C mutation in the Delta variant of SARS-CoV-2. Using reverse genetics, we isolated the G215C mutation in the WA1 strain of SARS-CoV-2 and demonstrate that it augments viral infection *in vitro* and *in vivo*. Mechanistically, we show that the G215C mutation increases RNA bound to N, the level of N in virions, and enlarges individual virion size. We also show evidence that the stably dimerized form of N remains even at excess concentrations of NEM; the results indicate that covalent bonds at least partially contribute to the N-N interaction in the context of viral infection. Together, our study shows that adaptation of the N protein in the Delta variant augments infection through increased encapsidation. These results have implications for understanding human infection by SARS-CoV-2 and adaptative mutations outside of the spike protein.

While the other reviewers were satisfied with the revised manuscript, Reviewer 2 had concerns about what we consider a minor mechanistic observation in the context of the findings in the manuscript. Covalent vs non-covalent N dimer formation has been the subject of several studies [Zhao *et al*, 2022 PNAS Nexus; Zhao *et al*, 2023 Science Advances; Zhao *et al* 2024 NAR]. Our work provides evidence that both covalent and non-covalent interactions contribute to stable dimer formation by the G215C mutation. While NEM treatment reduces the dimer abundance, it does not ablate formation, thus arguing for a covalent disulfide link via C215. Our results are consistent with two prior papers that found "*N:G215C forms a tightly bound, compact tetramer via disulfide cross-links of noncovalent dimers*" [Zhao *et al*, 2022 PNAS Nexus] and "*slightly enhanced self*

association under non-reducing compared to reducing conditions demonstrates contribution of disulfide bonds to the assembly" [Zhao *et al*, 2023 Science Advances]. Reviewer 2's concern focuses on the relative contribution of each and argue that non-covalent interactions are primarily responsible. Our data does not disagree nor detract from this argument; however, our data does show a covalent linkage plays a role in dimer formation.

While we have added data and modified the text to clarify, reviewer 2 has focused on this point in their comments following both the initial submission and revised manuscript. In their initial comments, they requested generation of a G215S SARS-CoV-2 mutant as well as the provided NEM experiments. In our prior email correspondence (May 21, 2024; August 7, 2024), we understood these to be essential experiments despite the limitations that we described. The experiments subsequently took 8 months and demonstrated that the N:G215S mutant had reduced encapsidation. In addition, the N:G215S mutant fails to replicate in primary human cells providing additional evidence for the importance of a covalent linkage. While reviewer 2 dismisses this effort, the study further demonstrates the importance of G215C in encapsidation and enhanced SARS-CoV-2 infection. These are the major finding of this study and highlight the importance of this N adaption for SARS-CoV-2 pathogenesis.

Reviewer 2 continues to focus on dimerization and covalent/non-covalent linkage in their comments on the revision. They imply the presence of the dimer seen in NEM-treated samples (Fig. 2C/D) could be further resolved by mass spectrometry to show they do in fact exist. They dismiss the difficulty of mass spec in this region using standard digests and indicate that we should identify, optimize, and test an alternative protocol that does not currently exist. However, even in this context, these experiments would still have to be conducted on post-lysis samples which the reviewer considered inadequate in the original manuscript. Given these constraints, it unclear how the proposed experiment would provide a definitive answer beyond both covalent/non-covalent linkages contribute to more stable N dimers in the context of G215C.

Overall, our manuscript shows that the G215C mutant in SARS-CoV-2 delta variant forms a more stable N dimer and augments viral infection *in vitro* and *in vivo*. Mechanistically, we show the G215C mutant has more encapsidation as measured by increased RNA binding to N, N incorporation into virions, and larger particles. Importantly, we show these enhancements are not observed in G215S mutant and data arguing that the response is partially linked to a covalent disulfide bond. Given the use of reverese genetics, in vitro and in vivo systems, and robust mechanistic data, we believe the manuscript is well suited for publication in Nature Communications.

Sincerely,

Emily Bruce, Ph.D

Dear Dr Bruce,

Thank you again for submitting your revised manuscript "Variant mutation in SARS-CoV-2 nucleocapsid enhances viral infection via altered genomic encapsidation" to Nature Communications. We have now received reports from the reviewers who evaluated the original version. On the basis of their comments (copied below), we have decided to invite an additional revision of your work.

You will see that, while the reviewers find that your revisions improved the manuscript, some important points remain to be addressed. Particularly reviewer #2 remains unconvinced by the new data relating to your main conclusion that disulfide bonds stabilize N-N dimers following mutation. We ask you to re-do the experiment presented in Figure 2, ensure equal loading of all samples, and provide a quantitative assessment of the data instead of a simple qualitative statement. Further, we ask you to better outline your issues with MS-based proof and temper too strong conclusions. Please revise your manuscript, addressing all the remaining issues raised by the reviewers.
[submission instructions omitted for space, I'm happy to forward the email if necessary]

Best regards,

Madlen Luckner
Editor
Nature Communications

REVIEWER COMMENTS

Reviewer #1 (Remarks to the Author):

Most issues are addressed in the revised manuscript, except for the comments 1 and 3.
I suggest a revision of the title, since the title did not point out the mutation G215C but the work mostly focus on one N mutation G215C.

We have now changed the title to 'Variant mutation G215C in SARS-CoV-2 nucleocapsid enhances viral infection via altered genomic encapsidation'.

The review work associated with the nucleocapsid mutations is still can be improved.

The manuscript has been modified to add additional data, analysis, and text changes.

Reviewer #2 (Remarks to the Author):

The manuscript by Kubinski et al provides very valuable information on the phenotype of the N:G215C mutation in vivo, and links this to enhanced nucleocapsid protein

oligomerization. Unfortunately in our view the conclusions linking the observed effects mechanistically to covalent cysteine crosslinks are still not completely convincing.

We appreciate the reviewers acknowledgment of "very valuable information" regarding the phenotype of the mutant and mechanistic insights. We believe the added data and revised text will clarify and ameliorate most of the reviewer concerns.

The additional Figures 2C/D show clearly that NEM does reduce the N dimer band: It seems obvious that the dimer band in lane 8 is fainter than 5 in Figure 2C, and that lanes 6-8 in Figure 2D are significantly fainter than lane 5. It appears there is bias in the interpretation of the magnitude of this effect in the authors' judgement. Our previous recommendation was that, in order to make this objectively clear, the authors scan the gels and provide quantitative estimates of how much dimer remains, similar to 2B. Just looking at 2D, my conclusion would be that NEM does indeed substantially reduce the dimer band, and that therefore possibly the majority of putatively formed disulfide bonds are post-lysis.

As requested, we have added an additional blot and quantified the change in dimer formation (Fig. S3). Examination confirms that the G215C mutant relative form a stable dimer relative to WT control virus. Treatment with NEM at time of lysis showed no consistent change in dimer formation across an escalating dose. Addition of NEM prior to lysis reduces dimer relative to control; however, dimer still consistently forms and is not responsive to an escalating dose. These data indicate that both covalent and non-covalent linkages govern N dimer formation in the context of G215C.

We have updated this portion of the results with the following language:
"When lysed in increasing concentrations of NEM (Fig 2C; 1-10 mM) the stably dimerized form was still present at similar levels to cells lysed without NEM (lanes 7,8 vs 6, see quantification in Fig S3). To further investigate whether the disulfide bonded dimer was present inside infected cells before lysis, we incubated cells with increasing concentrations of NEM before lysing cells in the same lysis buffer as above, including fresh NEM at the time of lysis (Fig 2D, Fig S3). While a ~80% reduction in dimerized N was observed, stable dimerized N was seen at every NEM dose including a ten-fold excess of typical concentrations. These results indicate covalent bonds contribute to N dimerization of the N:G215C mutant. While some of the linkages in the N dimer band could form post lysis, off target effects of NEM treatment and N dimer formation in late stage infection/assembly could also explain some of the reduction in prelysis treated samples. Similarly, while protein oligomers could be resistant to NEM similar to SDS resistant oligomers[30,31], sensitivity to DTT (Fig. 2A) argue against this possibility. In total, our data is consistent with both covalent and non-covalent interactions playing a role in the N-N interaction in the context of viral infection, driven by the cysteine residue at position 215." (Line 202-216).

FIG S3

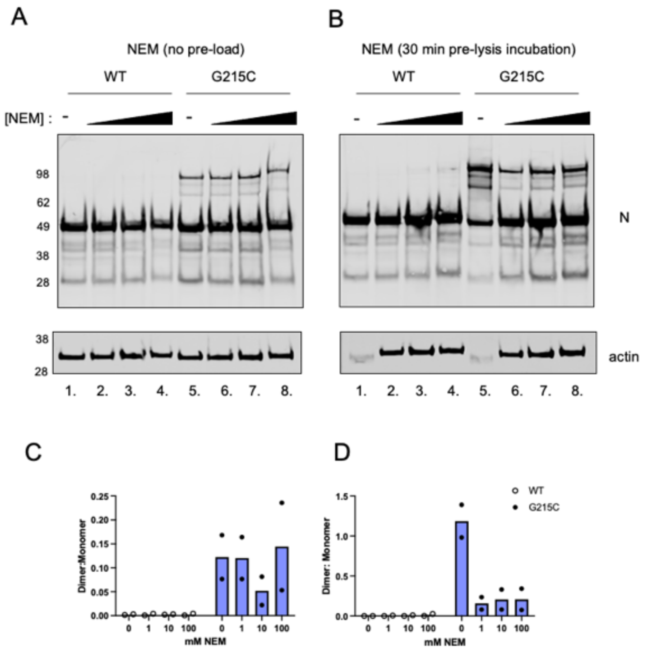

A related question is the possibility that even in the presence of non-covalent contributions of the cysteine to the oligomerization the oligomers could become partially SDS-resistant. It is not common but also not too unusual that protein oligomers can be SDS resistant, including coiled-coils [e.g. the SNARE complex (Nat Struct Mol Biol 5 (1998) 765), and HIV gp41 (JBC 10.1074/jbc.M110.101170)]. Based on the data this possibility cannot be ruled out and should be entertained.

We agree with the reviewers that partial resistance is “not common”, but does have some exceptions. However, our data with DTT argues against this possibility. As such, we have added the following text:

“Similarly, while protein oligomers could be resistant to NEM similar to SDS-resistant oligomers[30,31], sensitivity to DTT (Fig. 2A) argues against this possibility.” (Line 213-214).

As suggested previously, more unequivocal proof of covalent linkage could probably be obtained by mass spectrometry if applied to the exhaustively NEM-treated samples. The authors state they could not get good coverage in tryptic digests, but given the key role of this experiment I would suggest that alternative suitable MS protocols be tested, or details on why it could not be done should be discussed.

We argue that presence of the dimer in the context of NEM treatment pre-lysis is strong evidence that covalent linkage is intact. The request to identify, optimize, and subsequently test with alternative mass spectrometry protocols that do not currently exist is beyond the scope of the current manuscript. Importantly, these studies must utilize post-lysis samples and thus would have the same caveats the reviewer has outlined.

Another open question regarding the oligomeric state is whether any higher oligomers such as trimers occur. The authors point out that this is the case in other coronaviruses (p.4 line 192 in ms with comments). Quite unfortunately the way the gels in the present paper are run or shown does not allow to detect the presence or observe the absence of such larger oligomers. Is that from limited image display or could a different gel be run?

We apologize for lack of clarity as the full, uncropped gels were provided in the 'all westerns' file in the prior round of review. In general, we do not observe bands above the dimer, on a standard 4-12% gel. We have provided one of the uncropped gels below for clarity.

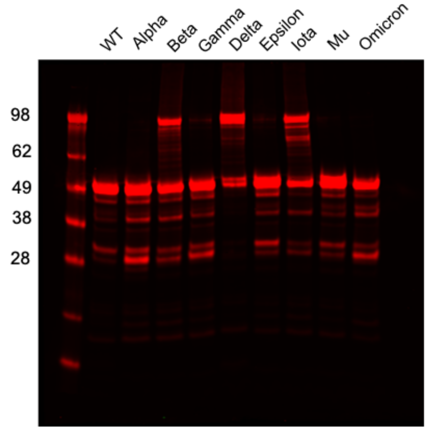

Figure 2: These membranes were probed with Sino Biological anti-N rabbit antibody (40143-R001 in red).

We appreciate the addition of the N:G215S experiments, and it is unfortunate that the G215S virus is unable to replicate. We concur with the authors' conclusion that additional critical interactions of this linker regions might be disrupted by the serine. This is very interesting in itself. Unfortunately, because of this interfering factor this 215S experiment does not add to the question regarding the relevance of covalent vs non-covalent interactions. How can the authors utilize this as evidence that it is covalent interactions causing the observed phenotype?

We thank the reviewer for acknowledging the difficulty of generating the N:G215S mutants and subsequent experiments that they requested.

While the lack of replication does not provide direct insight to covalent vs non-covalent interaction, it does provide relevant data to our findings. Our results demonstrate that the G215S mutant has reduced N packaging in virions contrasting increased encapsidation observed in the G215C mutant. The results imply that serine at this position does not compensate for cysteine despite a similar capacity to form non-covalent bonds. While the failure to replicate in primary cells precludes examination of the covalent/non-covalent bond question, the attenuation of G215S indicates that cysteine at this position is critical for the enhanced of SARS-CoV-2 infection. These findings provide significant support to our

main conclusion that the G215C mutation increases encapsidation in SARS-CoV-2 infection.

Page 8, line 327: Regarding Figure 3A, the authors' statement "beta did not consistently form ..." is strange, as the dimer band in the gel looks quite obvious. Can that be quantified, or is there additional data to support this?

The quantification of Figure 3A was provided in Figure 3B (located immediately to the right of 3A) in the prior round of review, as were all of the original western blots used to generate the quantification (in the 'allwesterns' file, attached again with this round of review and provided below for clarity).

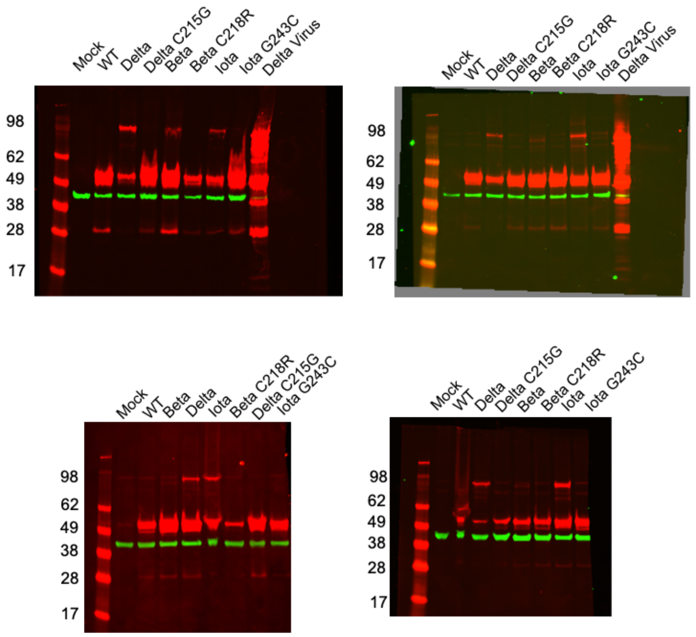

Figure 3 A/B: These membranes were probed with Sino Biological anti-N rabbit antibody (40143-R001 in red) and Novus Biologicals anti-B-actin mouse antibody (NB600-501 in green).

In the gels of Figure 3-6, were the samples NEM treated? Since Figure 2 shows a significant fraction (maybe majority) post-lysis formation, would that not similarly impact the samples in Figure 3, 4, 5, and 6? I assume these must have been NEM treated but this is not mentioned in the figure legend. If not, then how much of what we're seeing as a dimer band could have formed artificially post-lysis?

The figure legends are accurate as described in the text.

Other points:

* The legend of Figure 2 needs work. The panels and text seem mixed up. What are the lower sub-plots of panel 2C and 2D? There is no context and no Mw markers, and sub-panels seems to be cut out lanes?

The lower sub-plots of 2C and 2D are the actin loading control (as in Fig 3A, 3C, 4B, 4D, etc) and the molecular weight markers have been added to the C and D panels. We have revised the figure legend for clarity.

* New text p 6 line 263 (in ms with comments) refers to asterisks in 2A, but there are none.

The asterisk appears immediately to the right of the dimer band in 2A (outside top right corner of the blot). We have modified the figure legend to describe the asterisk in addition to the text.

* Figure S5C only shows a truncated gel cropped to the band for N, but it would be interesting to see the entire gel.

The “fullwesterns” file includes the requested full blots. We have also attached below for review.

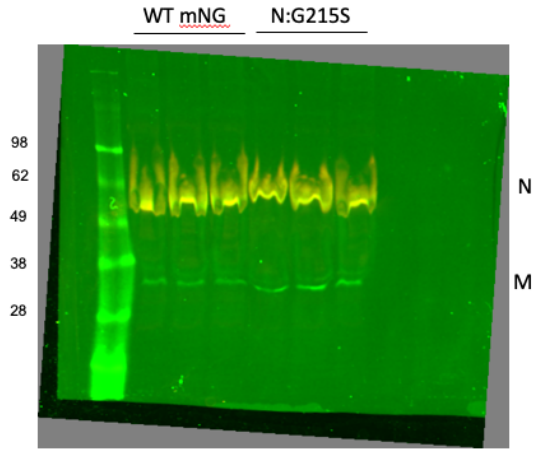

Fig S6: These membranes were probed with Sino Biological anti-N rabbit antibody (40143-R001 in red) and Cell Signaling anti-M mouse antibody (E5A8A in green). (Anti-mouse red secondary was also used on this blot, which is why the N band is yellow).

* p4 line 190: this sentence is incomplete
Corrected as requested.

* Figure S5A/B - the legend doesn't match the lines and data seems missing.

We have uploaded a revised figure with the correct data which is shown below. The green line was lost upon PDF conversion.

FIG S6

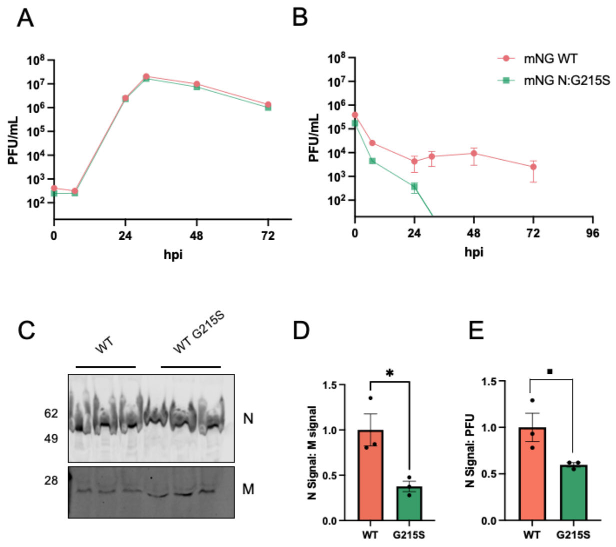

* p 14 line 507: As already previously pointed out, "a ~60 micromolar diameter" doesn't make sense. The correction of same error in the figure axis label has left off a closing parenthesis.

Revised as requested.

Reviewer #3 (Remarks to the Author):

Our comments have been adequately addressed, and we recommend publication.

We thank the reviewer for their time and effort in reviewing the revised manuscript, and are glad they feel the revision is ready for publication.

Reviewer #4 (Remarks to the Author):

I co-reviewed this manuscript with one of the reviewers who provided the listed reports. This is part of the Nature Communications initiative to facilitate training in peer review and to provide appropriate recognition for Early Career Researchers who co-review manuscripts.

We thank the reviewer for their time and effort in co-reviewing the manuscript.

Dear Dr Bruce,

Your manuscript entitled "Variant mutation G215C in SARS-CoV-2 nucleocapsid enhances viral infection via altered genomic encapsidation" has now been seen by 1 referee, whose comments are appended below. Considering their advice I regret to inform you that we cannot publish your manuscript in Nature Communications.

Although your work improved during revisions in terms of confirming the influence of mutation G215C on increased replication efficiency of SARS-CoV-2 in vitro and in vivo, the most relevant aspect for us to consider your work further, namely the mechanistic data explaining the increased stability of N dimers via a newly formed disulfide-bond, could not be strengthened sufficiently during revisions. Unfortunately, these reservations are sufficiently important to preclude publication of this study in Nature Communications.

However, I have consulted with Tobias Goris at Communications Biology, a selective open access journal from Nature Portfolio. They are very interested in your study and would in principle like to publish a suitably revised manuscript. They follow the arguments of R2 and ask that you address the remaining comments of the reviewers textually and in presentations. In particular, they ask that you please 1) tone down abstract and main text as the formation of disulfide bonds is not completely convincingly shown and 2) modify Fig 2 to contain the graphs from Fig S3D and 3) to react upon the criticism on Fig 3: Clearly show and critically discuss the large variations and uncertainties. Also we note that in general uncropped/raw blots/gels must be shown in the supplements to comply with our editorial requirements. If you would like to have your manuscript considered by the editors at Communications Biology, you may transfer the paper to them using the link provided below. Depending on the extent of the revisions made, they reserve the right to send the manuscript back for further peer review. If you would like to discuss your manuscript and the necessary revisions with an editor at Communications Biology, you can contact Tobias Goris directly at (tobias.goris@nature.com).

If you decide to submit your revised manuscript to Communications Biology, please ensure that your revision is accompanied by a detailed point-by-point response to all the reviewer comments and a cover letter summarizing the changes made to the manuscript. Please also include completed Life Sciences Reporting Summary and policy checklist forms and modify your manuscript if needed, to comply with the instructions in these documents.
Communications Biology is a fully open-access journal and an article processing charge will apply to any papers accepted for publication. For further details about Communications Biology, including their editorial policies and information about open access funding, please visit the journal website at https://www.nature.com/commsbio/.

We hope the Reviewers' comments, as well as the information on how to transfer your manuscript will be useful in helping you decide how to proceed. To transfer your manuscript please use our manuscript transfer portal. You will not have to re-supply

manuscript metadata and files, unless you wish to make modifications. For more information, please see our manuscript transfer FAQ page.

I am sorry that we cannot be more positive on this occasion and thank you for the opportunity to consider your work.

Best regards,

Madlen Luckner
Editor
Nature Communications

Reviewers' comments:

Reviewer #2 (Remarks to the Author):

As stated prominently in the abstract, the formation of disulfide bonds creating stable N-N dimers is a major mechanistic conclusion of the paper. Therefore this result should be established beyond a doubt.
Whether the increased N-N association occurs via a covalent or non-covalent linkage is not the key finding of our manuscript, which focuses on the consequences (to virus and host) of the G215C mutation, and the mechanism of action (increased encapsidation). To avoid over-stating our conclusions and address this reviewer's concern we have made the following modifications to the text.

We have modified the abstract, it now reads "*we identify a mutation (G215C), characteristic of the Delta variant, that introduces a novel cysteine into this linker domain, which results in the formation of a more stable N-N dimer*" (line 69-71) and we have removed any reference to a disulfide bond in the abstract. We have also added a line to the discussion "*While our data shows that this mutation increases N-N association in both a covalent and non-covalent manner, further work is needed to establish the relative roles of covalent vs non-covalent linkage at this residue.*" Line 387-389.

However, as we have pointed out in our prior comments, the biochemical methodology used is not very convincing. The control experiment for post-lysis formation Figure 2D shows that on average 80% of the observed putative covalent dimer is indeed artifactual post-lysis. It is good this is now quantified and stated in the manuscript. At the same time, this is a worrisome result, and for scientifically solid conclusions to be drawn from such experiments this problem needs to be faced head-on and transparently.

Unfortunately the authors have decided to continue relegating the quantitation into the

supplement, whereas we feel Figure S3D should go side-by-side with the other bar graphs in Figure 2B, so that readers clearly visually learn about this.
The control of post-lysis formation is actually Figure 2C, where NEM is added during lysis (and the quantification in this condition shows no consistant decrease in dimer formation). Figure 2D is an additional experiment we performed where we incubate infected cells in NEM for 30 minutes prior to lysis. This means that any disulfide bonds naturally formed in cells (say in virions assembling during this time) will also be blocked, so it is possible these conditions over-represent the fraction of covalent bonds that are artifactually formed. Due to space constraints we have not moved Figure S3C/D into Figure 2, but if required for publication this is an alteration we could make by shrinking the other subpanels.

Ordinarily one might be discouraged by such a strong artifactual disulfide formation, but the authors more optimistically rely on the remaining 20% to be a solid number. On the other hand, the authors dismiss the similar dimer populations of Beta in Figure 3B as unreliable. Is there any objective criterion for this judgement? To get clarity on this, it seems one would need to know the statistical variation of this 80%/20% post-lysis artifact vs intracellular disulfide across experiments.
We believe the Beta dimer formation is inconsistent because the P.1 Beta stock contained a mixed population at the 185 residue, so some infections were dominated by a R rather than a C. This was further supported by the fact that our P.2 Beta stock fully reverted, and the cysteine at position 185 was lost. This is why we focused on the G215C mutation, as it was the only one of the three maintained in the lineage.

The data in Figure S3C and D are from only two experiments, which showed a huge variation in the dimer/monomer ratio. For example without pre-load the dimer/monomer ratio seems to shift by a factor of 2 - 3. For the pre-lysis incubation of NEM, the two experiments carried out differ by a factor 2 - 4 in the two experiments, which means the average of 20% 'true' preexisting disulfide is not well determined (from n=2), and in one of the experiments seems to have been 10% or less?
The purpose of these experiments was primarily to provide justification for whether the more stable N-N dimer phenotype could be attributed to the individual point mutations, a necessary prerequisite to carrying out the reverse genetics experiment to rescue virus. We agree that more work is needed to comprehensively answer the question of what fraction of the N-N dimer occurs via covalent vs non-covalent linkages in infected cells during a SARS-CoV-2 infection, and the discussion now includes this point (line 387-389).

The authors point out that pre-lysis incubation with NEM might be problematic in itself, and we agree with that possibility, although there is no data to support this. At the same time, there could also be reasons why the control experiment is not perfect at revealing post-lysis disulfide bond creation. For example, could some intracellular compartments or microenvironments be protected from NEM? These are open questions, which reinforces the fact that this experimental design for detecting disulfide is at present not a sufficiently quantitative approach to draw conclusions from ~20% remaining disulfide bonds among ~80% artifactual post-lysis disulfide bonds.

As discussed in our prior round of review, if this were the case we would also expect these compartments to be shielded from DTT (which is the same weight as NEM), and treatment with DTT fully dissolves the ~98kDa dimer band that remains intact after lysis in the presence of NEM. Finally, in cells lysed in increasing concentrations of NEM we do not see a consistent drop in dimer formation. The 80/20 number is only when cells are incubated with NEM during infection, which could block the formation of *bona fide* covalent bonds as well as artifactual ones.

Furthermore, in the remaining paper the authors show in other figures dimer/monomer ratios from experiments apparently without precautions for post-lysis disulfide formation, which probably might be similarly 80% or more artifactual. It would be misleading not to point this out explicitly to the reader. Of course it would be better to repeat these experiments with NEM incubation pre-lysis.

The presence of NEM is clearly indicated in figure legends and the methods. Repeating additional experiments would not change the conclusions of the paper, given that we have numerous controls showing the dimerized form of N we observe is at least partially covalent.

Finally, the two image sections in Figure S6C should be shown as one contiguous gel (as in the rebuttal). Cropping everything in the gel but the lanes of interest and splitting them in separate images arranged as if it was one gel is unusual and questionable. The readers should have a chance to assess the quality of the experiment.

The two image sections in Figure S6C are from one contiguous gel, with each channel cropped to show the relevant protein, with no alternation in the loading order or omission of any samples. The full gel clearly shows that no lanes were omitted, as Reviewer 2 seems to be suggesting. While we fully agree that splicing lanes from different gels together is questionable, letter-boxing a section of gel containing the region surrounding a band of interest is standard practice, so we are quite confused as to why the Reviewer believes this is somehow suspect. Nonetheless, the full western blot of this experiment (and all the westerns) can be found in the attached 'kubinski_allwesterns' file, pasted below for ease of review.

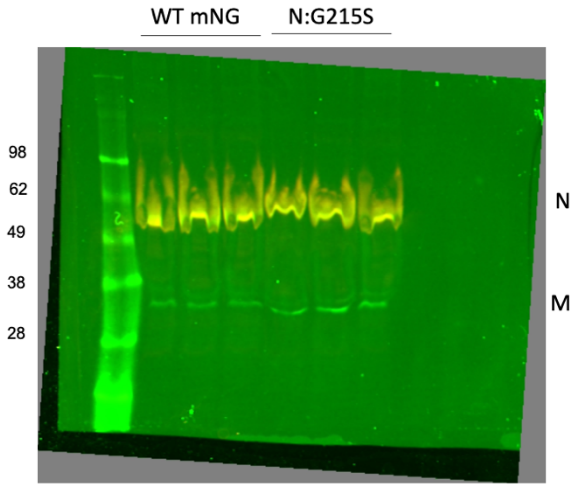

Fig S6: These membranes were probed with Sino Biological anti-N rabbit antibody (40143-R001 in red) and Cell Signaling anti-M mouse antibody (E5A8A in green). (Anti-mouse red secondary was also used on this blot, which is why the N band is yellow).

This email has been sent through the Springer Nature Tracking System NY-610A-NPG&MTS

Confidentiality Statement:

This e-mail is confidential and subject to copyright. Any unauthorised use or disclosure of its contents is prohibited. If you have received this email in error please

notify our Manuscript Tracking System Helpdesk team at http://platformsupport.nature.com .

Details of the confidentiality and pre-publicity policy may be found here http://www.nature.com/authors/policies/confidentiality.html

Privacy Policy | Update Profile
