# Supplementary material for: Variant mutation G215C in SARS-CoV-2 nucleocapsid enhances viral infection via altered genomic encapsidation
Source: PLoS Biol. 2025 Apr 29;23(4):e3003115. doi: 10.1371/journal.pbio.3003115 (PMC12040272; doi:10.1371/journal.pbio.3003115)
Supplement: Attachment — Submitted filename: Kubinski_Bruce_prior_reviews round3.pdf [file pbio.3003115.s012.pdf]

Dear Dr Bruce,

Your manuscript entitled "Variant mutation G215C in SARS-CoV-2 nucleocapsid enhances viral infection via altered genomic encapsidation" has now been seen by 1 referee, whose comments are appended below. Considering their advice I regret to inform you that we cannot publish your manuscript in Nature Communications.

Although your work improved during revisions in terms of confirming the influence of mutation G215C on increased replication efficiency of SARS-CoV-2 in vitro and in vivo, the most relevant aspect for us to consider your work further, namely the mechanistic data explaining the increased stability of N dimers via a newly formed disulfide-bond, could not be strengthened sufficiently during revisions. Unfortunately, these reservations are sufficiently important to preclude publication of this study in Nature Communications.

However, I have consulted with Tobias Goris at Communications Biology, a selective open access journal from Nature Portfolio. They are very interested in your study and would in principle like to publish a suitably revised manuscript. They follow the arguments of R2 and ask that you address the remaining comments of the reviewers textually and in presentations. In particular, they ask that you please 1) tone down abstract and main text as the formation of disulfide bonds is not completely convincingly shown and 2) modify Fig 2 to contain the graphs from Fig S3D and 3) to react upon the criticism on Fig 3: Clearly show and critically discuss the large variations and uncertainties. Also we note that in general uncropped/raw blots/gels must be shown in the supplements to comply with our editorial requirements. If you would like to have your manuscript considered by the editors at Communications Biology, you may transfer the paper to them using the link provided below. Depending on the extent of the revisions made, they reserve the right to send the manuscript back for further peer review. If you would like to discuss your manuscript and the necessary revisions with an editor at Communications Biology, you can contact Tobias Goris directly at (tobias.goris@nature.com).

If you decide to submit your revised manuscript to Communications Biology, please ensure that your revision is accompanied by a detailed point-by-point response to all the reviewer comments and a cover letter summarizing the changes made to the manuscript. Please also include completed Life Sciences Reporting Summary and policy checklist forms and modify your manuscript if needed, to comply with the instructions in these documents.
Communications Biology is a fully open-access journal and an article processing charge will apply to any papers accepted for publication. For further details about Communications Biology, including their editorial policies and information about open access funding, please visit the journal website at https://www.nature.com/commsbio/.

We hope the Reviewers' comments, as well as the information on how to transfer your manuscript will be useful in helping you decide how to proceed. To transfer your manuscript please use our manuscript transfer portal. You will not have to re-supply

manuscript metadata and files, unless you wish to make modifications. For more information, please see our manuscript transfer FAQ page.

I am sorry that we cannot be more positive on this occasion and thank you for the opportunity to consider your work.

Best regards,

Madlen Luckner
Editor
Nature Communications

Reviewers' comments:

Reviewer #2 (Remarks to the Author):

As stated prominently in the abstract, the formation of disulfide bonds creating stable N-N dimers is a major mechanistic conclusion of the paper. Therefore this result should be established beyond a doubt.
Whether the increased N-N association occurs via a covalent or non-covalent linkage is not the key finding of our manuscript, which focuses on the consequences (to virus and host) of the G215C mutation, and the mechanism of action (increased encapsidation). To avoid over-stating our conclusions and address this reviewer's concern we have made the following modifications to the text.

We have modified the abstract, it now reads "*we identify a mutation (G215C), characteristic of the Delta variant, that introduces a novel cysteine into this linker domain, which results in the formation of a more stable N-N dimer*" (line 69-71) and we have removed any reference to a disulfide bond in the abstract. We have also added a line to the discussion *"While our data shows that this mutation increases N-N association in both a covalent and non-covalent manner, further work is needed to establish the relative roles of covalent vs non-covalent linkage at this residue."* Line 387-389.

However, as we have pointed out in our prior comments, the biochemical methodology used is not very convincing. The control experiment for post-lysis formation Figure 2D shows that on average 80% of the observed putative covalent dimer is indeed artifactual post-lysis. It is good this is now quantified and stated in the manuscript. At the same time, this is a worrisome result, and for scientifically solid conclusions to be drawn from such experiments this problem needs to be faced head-on and transparently.

Unfortunately the authors have decided to continue relegating the quantitation into the

supplement, whereas we feel Figure S3D should go side-by-side with the other bar graphs in Figure 2B, so that readers clearly visually learn about this.
The control of post-lysis formation is actually Figure 2C, where NEM is added during lysis (and the quantification in this condition shows no consistant decrease in dimer formation). Figure 2D is an additional experiment we performed where we incubate infected cells in NEM for 30 minutes prior to lysis. This means that any disulfide bonds naturally formed in cells (say in virions assembling during this time) will also be blocked, so it is possible these conditions over-represent the fraction of covalent bonds that are artifactually formed. Due to space constraints we have not moved Figure S3C/D into Figure 2, but if required for publication this is an alteration we could make by shrinking the other subpanels.

Ordinarily one might be discouraged by such a strong artifactual disulfide formation, but the authors more optimistically rely on the remaining 20% to be a solid number. On the other hand, the authors dismiss the similar dimer populations of Beta in Figure 3B as unreliable. Is there any objective criterion for this judgement? To get clarity on this, it seems one would need to know the statistical variation of this 80%/20% post-lysis artifact vs intracellular disulfide across experiments.
We believe the Beta dimer formation is inconsistent because the P.1 Beta stock contained a mixed population at the 185 residue, so some infections were dominated by a R rather than a C. This was further supported by the fact that our P.2 Beta stock fully reverted, and the cysteine at position 185 was lost. This is why we focused on the G215C mutation, as it was the only one of the three maintained in the lineage.

The data in Figure S3C and D are from only two experiments, which showed a huge variation in the dimer/monomer ratio. For example without pre-load the dimer/monomer ratio seems to shift by a factor of 2 - 3. For the pre-lysis incubation of NEM, the two experiments carried out differ by a factor 2 - 4 in the two experiments, which means the average of 20% 'true' preexisting disulfide is not well determined (from n=2), and in one of the experiments seems to have been 10% or less?
The purpose of these experiments was primarily to provide justification for whether the more stable N-N dimer phenotype could be attributed to the individual point mutations, a necessary prerequisite to carrying out the reverse genetics experiment to rescue virus. We agree that more work is needed to comprehensively answer the question of what fraction of the N-N dimer occurs via covalent vs non-covalent linkages in infected cells during a SARS-CoV-2 infection, and the discussion now includes this point (line 387-389).

The authors point out that pre-lysis incubation with NEM might be problematic in itself, and we agree with that possibility, although there is no data to support this. At the same time, there could also be reasons why the control experiment is not perfect at revealing post-lysis disulfide bond creation. For example, could some intracellular compartments or microenvironments be protected from NEM? These are open questions, which reinforces the fact that this experimental design for detecting disulfide is at present not a sufficiently quantitative approach to draw conclusions from ~20% remaining disulfide bonds among ~80% artifactual post-lysis disulfide bonds.

As discussed in our prior round of review, if this were the case we would also expect these compartments to be shielded from DTT (which is the same weight as NEM), and treatment with DTT fully dissolves the ~98kDa dimer band that remains intact after lysis in the presence of NEM. Finally, in cells lysed in increasing concentrations of NEM we do not see a consistent drop in dimer formation. The 80/20 number is only when cells are incubated with NEM during infection, which could block the formation of *bona fide* covalent bonds as well as artifactual ones.

Furthermore, in the remaining paper the authors show in other figures dimer/monomer ratios from experiments apparently without precautions for post-lysis disulfide formation, which probably might be similarly 80% or more artifactual. It would be misleading not to point this out explicitly to the reader. Of course it would be better to repeat these experiments with NEM incubation pre-lysis.
The presence of NEM is clearly indicated in figure legends and the methods. Repeating additional experiments would not change the conclusions of the paper, given that we have numerous controls showing the dimerized form of N we observe is at least partially covalent.

Finally, the two image sections in Figure S6C should be shown as one contiguous gel (as in the rebuttal). Cropping everything in the gel but the lanes of interest and splitting them in separate images arranged as if it was one gel is unusual and questionable. The readers should have a chance to assess the quality of the experiment.
The two image sections in Figure S6C are from one contiguous gel, with each channel cropped to show the relevant protein, with no alternation in the loading order or omission of any samples. The full gel clearly shows that no lanes were omitted, as Reviewer 2 seems to be suggesting. While we fully agree that splicing lanes from different gels together is questionable, letter-boxing a section of gel containing the region surrounding a band of interest is standard practice, so we are quite confused as to why the Reviewer believes this is somehow suspect. Nonetheless, the full western blot of this experiment (and all the westerns) can be found in the attached 'kubinski_allwesterns' file, pasted below for ease of review.

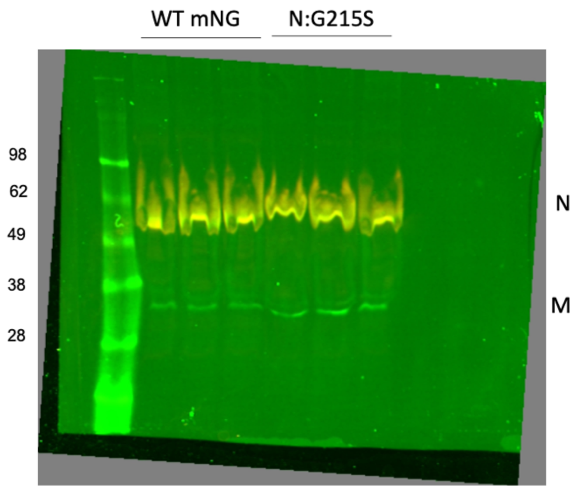

Fig S6: These membranes were probed with Sino Biological anti-N rabbit antibody (40143-R001 in red) and Cell Signaling anti-M mouse antibody (E5A8A in green). (Anti-mouse red secondary was also used on this blot, which is why the N band is yellow).

This email has been sent through the Springer Nature Tracking System NY-610A-NPG&MTS

Confidentiality Statement:

This e-mail is confidential and subject to copyright. Any unauthorised use or disclosure of its contents is prohibited. If you have received this email in error please

notify our Manuscript Tracking System Helpdesk team at http://platformsupport.nature.com .

Details of the confidentiality and pre-publicity policy may be found here http://www.nature.com/authors/policies/confidentiality.html

Privacy Policy | Update Profile
